# Identification of 39 stripe rust resistance loci in a panel of 465 winter wheat entries presumed to have high-temperature adult-plant resistance through genome-wide association mapping and marker-assisted detection

**DOI:** 10.3389/fpls.2024.1514926

**Published:** 2025-01-07

**Authors:** Fangjie Yao, Meinan Wang, Deven R. See, Ennian Yang, Guoyue Chen, Xianming Chen

**Affiliations:** ^1^ Environment Friendly Crop Germplasm Innovation and Genetic Improvement Key Laboratory of Sichuan Province, Key Laboratory of Wheat Biology and Genetic Improvement in Southwestern China, Crop Research Institute, Sichuan Academy of Agricultural Sciences, Chengdu, Sichuan, China; ^2^ Department of Plant Pathology, Washington State University, Pullman, WA, United States; ^3^ US Department of Agriculture, Agricultural Research Service, Wheat Health, Genetics, and Quality Research Unit, Pullman, WA, United States; ^4^ Triticeae Research Institute, Sichuan Agricultural University, Chengdu, Sichuan, China; ^5^ State Key Laboratory of Crop Gene Exploitation and Utilization in Southwest China, Sichuan Agricultural University, Chengdu, Sichuan, China

**Keywords:** wheat, stripe rust, durable resistance, genome-wide association study, marker-assisted detection

## Abstract

Stripe rust of wheat is a serious disease caused by *Puccinia striiformis* f. sp. *tritici* (*Pst*). Growing resistant cultivars is the most preferred approach to control the disease. To identify wheat genotypes with quantitative trait loci (QTL) for durable resistance to stripe rust, 465 winter wheat entries that were presumed to have high-temperature adult-plant (HTAP) resistance were used in this study. In the greenhouse seedling tests with seven *Pst* races, 16 entries were resistant to all the tested races. The 465 entries were also phenotyped for stripe rust responses at the adult-plant stage under natural infection of *Pst* in multiple field locations from 2018 to 2021 in the Washington state, and 345 entries were found to have stable resistance. The contrast of the susceptibility in the greenhouse seedling tests and the resistance in the field adult-plant stage for most of the entries indicated predominantly HTAP resistance in this panel. The durability of the resistance was demonstrated by a subset of 175 entries that were tested in multiple locations from 2007 to 2021. The 465 entries were genotyped through genotyping by multiplexed sequencing of single-nucleotide polymorphism (SNP) markers. Combining the stripe rust response and SNP marker data, a genome-wide association study (GWAS) was conducted, resulting in 143 marker–trait associations, from which 28 QTL that were detected at least with two races or in two field environments were identified, including seven for all-stage resistance and 21 for HTAP resistance. These QTL each explained 6.0% to 40.0% of the phenotypic variation. Compared with previously reported *Yr* genes and QTL based on their genomic positions, five QTL including two for HTAP resistance were identified as new. A total of 10 user-friendly Kompetitive allele specific PCR (KASP) markers were developed for eight of the HTAP resistance loci. In addition, molecular markers were used to detect 13 previously reported HTAP resistance genes/QTL, including two also identified in the GWAS analyses, and their frequencies ranged from 0.86% to 88.17% in the panel. The durable resistant genotypes, the genes/QTL identified, and the KASP markers developed in this study should be useful to develop wheat cultivars with long-lasting resistance to stripe rust.

## 1 Introduction

Stripe rust, also called yellow rust (*Yr*), caused by *Puccinia striiformis* f. sp. *tritici* (*Pst*), is an important disease of wheat worldwide (Stubbs, 1985; Chen, 2005, 2020). In the United States, wheat stripe rust caused damages mostly in the states west of the Rocky Mountains before 2000 and has become a serious problem throughout the inland US since 2000 (Line, 2002; Chen, 2005, 2007; Chen and Kang, 2017). The disease can cause huge damage in terms of yield losses and/or cost of fungicide application—for example, yield losses of 10%–30% were reported in Washington and/or Idaho, Montana, and Oregon in 1959–1961, 1974, 1976, and 1980. The worst stripe rust epidemic in the western United States was in 1981, which severely damaged over 70% of the wheat acreages in these Pacific Northwest states and led to the first widespread use of chemicals to reduce yield losses but still caused more than one million tons of yield loss, including 10% yield loss in the Washington state alone (Line, 2002; Chen, 2020). Although the yield losses in these years were very high, they counted for only 0.84% (1959) to 2.17% (1976) of the national wheat production, as the disease occurred mainly in the western states. Whenever the Great Plains and the eastern states had severe stripe rust, the national yield losses were much higher. The national potential yield losses reached 3.66% (including 25% in California and 10% in Kansas and Nebraska) in 2003, 4.16% (including 10% in Kansas and Texas) in 2010, 8.7% (including 25% in Oklahoma and 15% in Kansas, South Dakota, and Minnesota) in 2015, and 5.61% (including 18% in Oklahoma and 9% in Kansas) in 2016 or 2,421,121, 2,608,720, 4,509,149, and 3,526,926 metric tons, respectively (Chen, 2020). Although the actual yield losses were much reduced from these estimates, the reductions of the yield losses by fungicide applications cost millions of dollars in each year.

To control stripe rust, the best approach is to develop and plant resistant cultivars, especially cultivars combining effective all-stage resistance (ASR) and high-temperature adult-plant (HTAP) resistance to achieve high-level and durable resistance (Chen, 2013; Liu et al., 2018, 2020). ASR is also known as seedling resistance as it can be detected at the seedling stage but expressed throughout all growth stages. It typically shows high-level resistance when effective and is not affected much by temperatures and rust pressure. As ASR is usually controlled by single genes and qualitatively inherited, it can be easily incorporated into new cultivars. However, ASR is mostly race-specific and vulnerable to new virulent races of the pathogen. In contrast, HTAP resistance starts expressing or increases the level of resistance when the weather becomes warm, and the plants passes the seedling stage. This type of resistance is usually controlled by quantitative trait locus or loci (QTL) and less easy for use in breeding programs than ASR. HTAP resistance is usually partial, and the resistance level can be affected by temperature, growth stage, and rust pressure, and as such, HTAP resistance may not be adequate for the complete protection of the crop (Chen, 2013; Liu et al., 2019a, b; Li et al., 2023, 2024). When cultivars do not have adequate resistance, fungicide application is needed to control a disease (Chen, 2014; Liu et al., 2019a). Therefore, the best strategy is to develop wheat cultivars with pyramided genes for high-level HTAP resistance or combined both ASR and HTAP resistance to increase the level and durability of resistance (Chen, 2013, 2020; Liu et al., 2018, 2019).

Up to now, 87 permanently named *Yr* genes and hundreds of temporarily named genes or QTL have been reported for stripe rust resistance in wheat (Wang and Chen, 2017; Feng et al., 2023; Zhu et al., 2023; Sharma et al., 2024). Among the 87 named *Yr* genes, 60 were ASR genes and 27 were adult-plant resistance (APR) or HTAP resistance genes, and 13 were cloned (Wang and Chen, 2017; Long et al., 2024; Sharma et al., 2024; Zhou et al., 2024). Breeders prefer genes already present in high-yielding and adapted wheat cultivars. Unfortunately, with the emergence of new *Pst* races, many resistance genes widely deployed in wheat cultivars have been overcome. Among the 60 ASR genes, only a few, such as *Yr5*, *Yr15*, *Yr64*, and *Yr65*, are still effective against the *Pst* populations in the United States and other countries (Cheng et al., 2014; Qie et al., 2019; Wan and Chen, 2014; Wan et al., 2016; Chen et al., 2021; Wang et al., 2022). Many mapped genes or QTL for APR or HTAP resistance have small and variable effects. Often because their effects are too small or too variable, minor genes or QTL are too difficult to be used in breeding programs. Genes or QTL with strong effects for durable type of resistance like HTAP resistance are more useful in breeding programs. Therefore, the identification of new genes for effective ASR and high-level HTAP resistance is essential to develop wheat cultivars with adequate and durable resistance.

Genome-wide association study (GWAS) has been widely used to identify marker–trait associations (MTAs) for various traits in major crops, including wheat resistance to stripe rust. Several studies on stripe rust resistance in wheat germplasm using the GWAS approach have been reported (Maccaferri et al., 2015; Bulli et al., 2016; Liu et al., 2017a, b, c; Liu et al., 2019c, 2020; Mu et al., 2020; Yao et al., 2020; Khan et al., 2024; Marone et al., 2024). These studies used various panels of germplasms including spring and winter hexaploid wheat (*Triticum aestivum*), durum wheat (*T. turgidum* ssp. *durum*), emmer wheat (*T. turgidum* ssp. *dicoccum*), and other tetraploid wheats (*T. turgidum* ssp. ssp. *turanicum*, spp. *turgidum*, ssp. *polonicum*, ssp. *carthlicum*, ssp. *dicoccum*, and ssp. *dicoccoides*). Many QTL for response to stripe rust have been identified, but only a few were well characterized as for durable resistance. From a winter wheat panel of world wheat germplasm collection, Maccaferri et al. (2015) detected a locus for APR on the short arm of chromosome 6B (*QYr.ucw-6B*) in several wheat germplasm accessions. This locus was validated through molecular mapping using several bi-parental populations and permanently named as *Yr78* (Dong et al., 2017). Yao et al. (2021) detected five novel stripe rust resistance loci using a panel of Chinese wheat landraces. Using a spring wheat panel and a winter wheat panel consisting of cultivars, breeding lines, and genetic stocks from various wheat production regions in the United States, Liu et al. (2020) and Mu et al. (2020) identified 37 and 51 loci for resistance to stripe rust, respectively, including 10 novel loci in each of the studies, and determined different genes for different types of resistance used in different regions. These studies demonstrated that GWAS is a powerful approach to detect genes in many germplasm accessions and to identify new genes for stripe rust resistance.

In the present study, we identified stripe rust resistance loci in a panel of 465 winter wheat entries using the GWAS approach. The nursery of these entries was previously tested for many years to characterize stripe rust resistance, and the data indicated that most of the entries have HTAP resistance. Our objectives of this study were (1) to evaluate the stripe rust resistance of the 465 entries by testing them at the seedling stage with various *Pst* races and at the adult-plant stage in the fields under natural infections to identify entries with durable resistance under multiple environments, (2) to conduct genome-wide association mapping using genome-wide SNP markers to identify loci associated with stripe rust responses, (3) to identify new resistance loci by comparing the loci identified in the present study with previously reported loci, and (4) to convert SNP markers for some of the HTAP resistance loci into Kompetitive allele-specific PCR (KASP) markers to be used in wheat breeding programs. The wheat genotypes identified to have durable HTAP resistance, the identified resistance QTL, and the developed KASP markers should be useful in breeding programs to develop new wheat cultivars with adequate and durable resistance to stripe rust.

## 2 Materials and methods

### 2.1 Plant materials

A panel of winter wheat (*T. aestivum*) entries was assembled by selecting genotypes (genetic stocks, landraces, cultivars, and breeding lines) presumably with HTAP resistance from our initial screening of wheat germplasm for stripe rust resistance in the greenhouse and fields and started being tested in the fields as a winter high-temperature adult-plant resistant wheat nursery (WHAN) since 2007 (https://striperust.wsu.edu). The majority of the entries were previously tested in the greenhouse at both the seedling stage under the low-temperature profile (diurnal temperature gradually changing from 4°C at 2:00 p.m. to 20°C at 2:00 p.m.) and the adult-plant stage under the high-temperature profile (diurnal temperature gradually changing from 10°C at 2:00 p.m. to 30°C at 2:00 p.m.) (Chen, 2013). The number of entries in the panel was increased over the years and fixed to 465 for testing with recently predominant races of *Pst* at the seedling stage in the greenhouse and at the adult-plant stage in the fields from 2018 to 2021 to facilitate the GWAS analysis. Spring wheat ‘Avocet S’ (AvS), which is highly susceptible throughout the growth stages, and winter wheat ‘Nugaines’, which is highly susceptible in the seedling stage, were used for the reproduction of urediniospores of selected *Pst* isolates in the greenhouse. Winter wheat ‘PS 279’, which is highly susceptible throughout the growth stages and does not have any known genes for resistance to stripe rust, was used as a susceptible check in the greenhouse tests and the field tests and as a spreader surrounding the fields to create a uniform and high-level stripe rust pressure. The 18 *Yr* single-gene lines, which are used to differentiate *Pst* races (Wan and Chen, 2014; Wan et al., 2016; Wang et al., 2022), were used to confirm the races of the isolates used in the greenhouse tests. Information on the 465 entries is provided in **Supplementary Table S1**.

### 2.2 *Pst* races

Seven *Pst* races—PSTv-4, PSTv-14, PSTv-18, PSTv-37, PSTv-40, PSTv-51, and PSTv-198—which were predominant in the United States over the past decade except PSTv-51 that has the broadest virulence spectrum (Wan and Chen, 2014; Wan et al., 2016; Wang et al., 2022), were selected to phenotype the 465 entries for stripe rust responses at the seedling stage in the greenhouse. PSTv-37 is the most widely distributed and most frequent race throughout the United States. PSTv-4, PSTv-14, and PSTv-40 are popular races in the US Pacific Northwest. PSTv-198 is also an epidemic race with significant frequencies in some years. PSTv-18 is the oldest race but has occurred in the Pacific Northwest with a significant frequency almost every year. This race is avirulent to all *Yr* single-gene differentials but virulent to some wheat cultivars/lines like Nugaines and PS 279. The virulence/avirulence formulae of the seven races on the 18 *Yr* single-gene differentials and the isolates used to represent these races are given in **Supplementary Table S2**. Compared to the *Yr* gene symbols used in Wan and Chen (2014), *YrTye* was replaced by *Yr76* and *YrTr1* was replaced by *Yr85* as these genes were permanently named by Xiang et al. (2016) and Feng et al. (2023), respectively.

### 2.3 Seedling tests in the greenhouse

The 465 wheat entries were evaluated at the seedling stage for stripe rust responses under controlled greenhouse conditions. The seven races (PSTv-4, PSTv-14, PSTv-18, PSTv-37, PSTv-40, PSTv-51, and PSTv-198) were used separately in the tests. The urediniospores of each race were increased using Nugaines or selected *Yr* single-gene differentials (**Supplementary Table S2**) and confirmed for race identity by testing the reproduced urediniospores on the set of 18 *Yr* single-gene differentials (Wan and Chen, 2014; Wan et al., 2016). Five to seven seeds of each entry and susceptible check AvS were planted in each well of plastic trays filled with a soil mixture and grown under the previously described conditions (Chen et al., 2002). After having been grown for 10 days to the two-leaf stage, the seedlings were uniformly dust-inoculated with a mixture of urediniospores and talc at a 1:20 ratio. After incubating in a dew chamber at 10°C for 24 h in the darkness, the seedlings were transferred to a growth chamber set at a diurnal cycle changing from 4°C at 2:00 a.m. to 20°C at 2:00 p.m. and 8-h dark/16-h light (Chen and Line, 1992). Infection type (IT) was recorded 18–20 days after inoculation based on the 0–9 scale (Line and Qayoum, 1992) when *Pst* was fully sporulating on the susceptible check.

### 2.4 Adult-plant tests in the fields

The 465 entries were evaluated from 2018 to 2021 for stripe rust response in the fields at Mount Vernon (48°25′12″ N, 122°19′34″ W) in the northwestern Washington and Palouse Conservation Field Station (PCFS, 46°43′59″ N, 117°10′00″ W) and Spillman Farm (SP, 46°43′47.1972″ N, 117°10′54.2568″ W) near Pullman in the southeastern Washington, referred to as year–location environments 18-21MV, 18-20PCFS, and 19-20SP, respectively. As the nursery was assembled and tested over the years, 175 of the 465 entries were also tested for stripe rust responses in the three locations from 2007 to 2017, referred to as environments 07-17MV, 07-17PCFS, and 08-17SP, respectively. In each environment, each entry of the nursery was planted in a single row of 50 cm long and 30 cm between rows in late October or early November. PS 279 was planted after each 20 rows and around the plots as a susceptible check and stripe rust spreader. The field tests were conducted under rainfed conditions without irrigation. All field tests were under the natural infection of *Pst*, except the Pullman locations in 2014, 2019, and 2021, where the nursery was inoculated before the boot stage with talc mixture of urediniospores collected from the same field in the previous years and stored in liquid nitrogen. The infection type and disease severity (DS) were recorded when the susceptible check PS 279 was fully infected with DS of 80%–100%. DS was assessed visually as the percentage of infected leaf area (0%–100%). To improve the normality of the DS data, relative DS (rDS) was calculated using the formula: rDS (%) = DS/DS_PS279_ × 100, where DS_PS279_ is the average DS value of PS 279 in the same environment. IT was recorded based on the 0–9 scales as described in Line and Qayoum (1992).

### 2.5 Phenotypic data analyses

For all tests, including the 465 entries tested in the recent nine environments and with the seven *Pst* races and the 175 entries tested in 32 environments, violin plots were drawn to show the distribution of the IT and rDS data using the *ggplots* package in R V3.6.2 (Wickham, 2016). The best linear unbiased estimate (BLUE) was calculated for the IT and rDS data separately assuming fixed effects for genotypes using IciMapping V4.0 (Meng et al., 2015). The minimum, maximum, and mean values, standard deviations, and coefficient of variation of stripe rust responses were calculated using EXCEL (Microsoft, Redmond, WA, USA). Pearson’s correlation coefficients (*r*) of pairwise environments were calculated and graphed using the *corrplot* package in R V3.6.2. Broad-sense heritability (*H* ^2^) was estimated using SAS V8.0 (SAS Institute Inc., Cary, NC, USA) and the formula: *H* ^2^ = σ^2_G_^/[σ^2_G_^ + (σ^2_E_^ + σ^2_E×G_^ + σ^2e^)/n], where σ^2_G_^ is the variance of genotypes, σ^2_E_^ is the variance of environments, σ^2_G×E_^ is the variance of the interaction between genotype and environment, σ^2_e_^ is the variance of residuals, and *n* is the number of environments. Genotype, environment, and the genotype×environment interactions were treated as random factors.

### 2.6 Genotyping

A leaf sample was collected from one single seedling plant for each entry grown in the greenhouse, and genomic DNA was extracted from the sample using the cetyltrimethyl ammonium bromide method (Stewart and Via, 1993). The DNA concentration was determined using a BioTek Synergy 2 Microplate reader (BioTek, Winooski, VT, USA) and diluted to the concentration of 20 ng/μL. The 465 entries were genotyped using genotyping by multiplex sequencing (GMS) on an Ion Proton system (Life Technologies Inc., Carlsbad, CA, USA) based on the protocol developed by Ruff et al. (2020). In addition, one sequence-tagged site (STS) marker, 10 simple nucleotide repeat (SSR) markers, and nine KASP markers, which are closely linked to or diagnostic of 13 previously reported *Yr* genes or QTL for HTAP resistance, were used to genotype the 465 winter wheat entries. The 13 genes/QTL were *Yr16* (Agenbag et al., 2012), *Yr17* (Liu et al., 2018), *Yr18* (Lagudah et al., 2009), *Yr30* (Spielmeyer et al., 2003), *Yr36* (Uauy et al., 2005), *Yr46* (Forrest et al., 2014), *Yr52* (Ren et al., 2012), *Yr59* (Zhou et al., 2014b), *Yr62* (Lu et al., 2014), *Yr78* (Dong et al., 2017), *Qyr.wgp-1B.1* (Naruoka et al., 2015), and *QYrsk.wgp-3BS* and *QYrsk.wgp-4BL* (Liu et al., 2019b). The markers for race-specific ASR gene *Yr17* were included as the gene is linked to the HTAP resistance locus *YrM1225* (Liu et al., 2018; Li et al., 2023). These markers, their representing *Yr* genes or QTL, and the primer sequences are provided in **Supplementary Table S3**.

Polymerase chain reaction (PCR) amplifications and KASP assays were conducted as described in Liu et al. (2019a, 2020). The PCR products of SSR and STS were detected using an ABI3730 DNA fragment analyzer (Applied Biosystems, Grand Island, NY, USA), and those of the KASP markers were detected using a Light-Cycler 480 real-time PCR system (Roche Applied Science, Indianapolis, IN, USA). The SSR alleles were scored using software GeneMarker v2.2.0 (Soft Genetics, State College, PA, USA).

### 2.7 Genetic diversity and population structure

After quality control, the polymorphism markers with missing rate ≤50% and minor allele frequency (MAF) ≥0.05 were used for genetic diversity and population structure analyses. Genetic diversity was estimated using Power-Marker V3.25 (Liu and Muse, 2005). Population structure was analyzed using software STRUCTHRE V2.3.4 with the Bayesian clustering algorithm (Pritchard et al., 2000) and using the parameter settings described in Yao et al. (2020). The optimum number (K) of subpopulations was determined using the software STRUCTURE HARVESTER (http://taylor0.biology.ucla.edu/structureHarvester/) (Evanno et al., 2005).

### 2.8 Linkage disequilibrium

To determine the interval defining a QTL and compare it with the previously reported *Yr* genes and QTL on the integrated map, the linkage disequilibrium (LD) decay distance was estimated as squared allele frequency correlation coefficient (*r* ^2^) between intra-chromosomal marker pairs using the software Tassel V3.0 (Bulli et al., 2016). All SNP markers with known chromosomal positions were used to calculate the LD decay distance. To estimate the LD distance, a locally weighted polynomial regression (LOESS)-based curve was fitted on the scatter plot using the intra-chromosomal pairwise *r* ^2^ values against the genetic distance. The genetic distance at which the LD decay curve intersects with the critical value *r* ^2^ = 0.1 was used as the threshold to determine the confidence interval of significant QTL (Yao et al., 2020).

### 2.9 Genome-wide association study

Two association analyses were conducted using the Genome Association and Prediction Integrated Tool (GAPIT) R package version 2.0 (Tang et al., 2016). The first GWAS analysis was performed using the adult-plant stage IT and rDS data of the 465 entries in 10 field environments (including the BLUE values) and the seedling IT data of the greenhouse tests with the seven races. The second GWAS analysis was conducted using the IT and rDS data of the 175 entries in 32 field environments from 2007 to 2017 and the BLUE values across these environments. The genotype data used for the two GWAS analysis were filtrated according to the number of entries with the criteria of missing rate <50% and MAF >0.05. To reduce the false positive associations from the type I error, a mixed linear model (MLM) (Yu et al., 2006) with both kinship (K) and population structure (Q) matrices as covariates were used for the GWAS analyses of both the 465 and 175 entries. Loci that were significant in at least two of the field environments or of the race tests or located within the confidence intervals of LD decay (*r* ^2^ ≤ 0.1) were considered as one QTL. Manhattan plots were drawn using the *CMplot* package in R V3.6.2 (https://github.com/YinLiLin/CMplot).

### 2.10 Comparison of QTL identified in the present study with previously reported *Yr* genes or QTL

The QTL detected in the present study were compared with previously reported *Yr* genes and QTL in the integrated map (Maccaferri et al., 2015; Wang et al., 2014). The positions of the polymorphic SNPs identified through GMS in the present study were 100% in agreement with their positions in the integrated map generated with the 90K SNP chip data. Therefore, we were able to compare the positions of our QTL with the previously reported *Yr* genes and QTL. With the LD decay distance (*r* ^2^ ≤ 0.10) as the interval distance, we considered our QTL the same as a previously reported gene/QTL if the marker(s) associated with our QTL were mapped to the same interval of the previous one.

### 2.11 Developing new KASP markers for HTAP resistance QTL identified in the present study

After HTAP resistance QTL were mapped through GWAS, KASP markers were developed for the QTL with relatively large effects following the methods described in Liu et al. (2018). Their SNP markers were converted to KASP markers using the primer sequences of CerealsDB (http://www.cerealsdb.uk.net/cerealgenomics/CerealsDB/KASP_primers_for_iSelect.php). The primers were synthesized by Sigma-Aldrich (St. Louis, MO, USA). The KASP markers were tested on the 465 entries for validation following the method described above.

### 2.12 Determining the frequencies of resistance gene/QTL

The frequencies of the resistance genes or QTL in the panel of 465 entries were determined using their markers. To reduce false positives, entries that had the resistance allele but were as susceptible as the susceptible check were not considered to have the gene/QTL (Liu et al., 2020; Mu et al., 2020).

## 3 Results

### 3.1 Stripe rust resistance and broad-sense heritability

**Figure 1:**
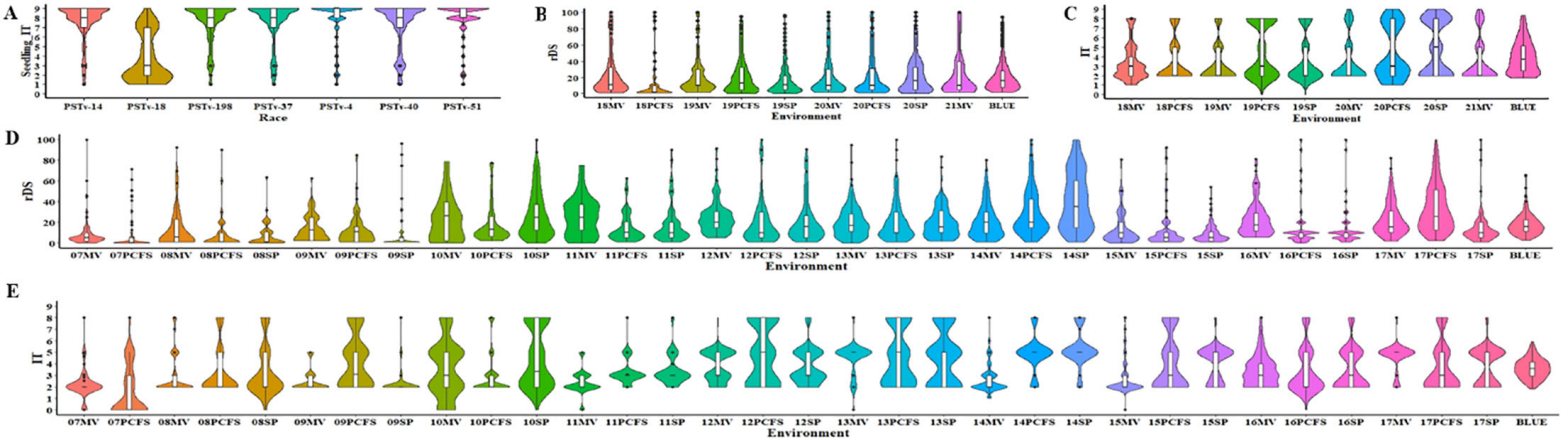
Violin plots showing the stripe rust response distributions in the winter wheat panel. **(A)** Infection types (IT) of 465 entries of the whole panel tested with seven races of *Puccinia striiformis* f. sp. *tritici* in the seedling stage at the low-diurnal-temperature cycle (4°C –20°C) in the greenhouse. **(B)** Relative disease severity (rDS) values and **(C)** IT values of the 465 entries in the whole panel at the adult-plant stage tested in different environments (year and location) from 2018 (18) to 2021 (21) at Mount Vernon (MV) in western Washington and Palouse Conservation Farm Station (PCFS) and Spillman Farm (SP) near Pullman in eastern Washington. **(D)** rDS values and **(E)** IT values of 175 entries of a sub-panel at the adult-plant stage in various environments from 2007 (07) to 2017 (17). BLUE, best linear unbiased estimator across all field environments.

The 465 winter wheat entries were tested at the seedling stage with seven *Pst* races (PSTv-4, PSTv-14, PSTv-18, PSTv-37, PSTv-40, PSTv-51, and PSTv-198) and at the adult-plant stage in nine field environments (18MV, 18PFS, 19MV, 19PCFS, 19SP, 20MV, 20PFS, 20SP, and 21MV). Most of the entries were susceptible in the greenhouse seedling tests but showed resistance at the adult-plant stage in the field tests (**Figure 1**). Except for the seedling test with the least virulent race, PSTv-18, the majority of entries (306–375 entries; 65.81%–80.65%) were highly susceptible (IT 8-9) in the seedling tests with different races (**Figure 1A**, **Table 1**; **Supplementary Table S1**). Of the 465 entries, 16 (3.44%) were resistant to all seven races, indicating that they have genes or gene combinations effective against the diverse *Pst* races (**Table 1**; **Supplementary Table S1**). In contrast, most of the 465 entries were resistant at the adult-plant stage in the fields, as 345 entries (74.19%) had BLUE_IT ≤5 and 428 (92.04%) had BLUE_rDS ≤50%. The contrast responses indicated that most of the entries have various levels of HTAP resistance. Combining the BLUE_IT and BLUE_rDS results, 345 entries (74.19%), including the 16 entries with ASR effective against all races tested in the seedling stage, showed stably moderate to high levels of resistance across the nine field environments (**Figures 1B, C**; **Table 1**). These entries with effective ASR and/or high level of HTAP resistance could be used in breeding programs.

**Table 1:** Distribution of stripe rust responses and QTL detected from the greenhouse seedling tests and in adult-plant stage from the field environments for the 465 or 175 wheat entries.

Test	Race or field environment^a^	No. of HR entries	No. of MR entries	No. of MS entries	No. of HS entries	No. of HR entries	No. of MR entries	No. of MS entries	No. of HS entries	No. of MTAs	QTL^b^
		IT 0–3	IT 4–5	IT 6–7	IT 8–9	rDS <25%	rDS 25–50%	rDS 50–75%	rDS >75%		
Seedling tests for 465 entries	PSTv-14	40	26	51	348	–	–	–	–	3	1B, 6A.1, 6A.2
	PSTv-18	275	58	81	51	–	–	–	–	0	–
	PSTv-198	47	29	60	329	–	–	–	–	1	6D.3
	PSTv-37	59	22	47	337	–	–	–	–	1	1B
	PSTv-4	59	17	26	363	–	–	–	–	2	1B, 6A.1
	PSTv-40	78	28	53	306	–	–	–	–	3	6A.1, 6D.1, 7A.2
	PSTv-51	37	12	41	375	–	–	–	–	4	5D, 6A.1, 6A.2, 6D.3
Adult-plant tests for 465 entries	18MV	318	86	22	39	327	55	33	50	5	2A.1, 2A.2, 4D, 6A.3
	18PCFS	310	80	0	75	422	23	0	20	9	2A.1, 2A.2, 3B, 4D, 6A.3
	19MV	334	76	1	54	326	92	12	35	9	2A.1, 2A.2, 2B, 4D, 6D.2
	19PCFS	235	47	2	181	326	76	38	25	4	5B.1, 5B.2, 7B.1
	19SP	308	59	1	97	364	66	28	7	3	2A.1, 2A.2
	20MV	327	60	1	77	327	79	29	30	3	2A.2, 4D,6A.3
	20PCFS	269	23	55	118	323	55	44	43	2	6D.2
	20SP	210	69	0	186	298	115	33	19	4	–
	21MV	314	70	0	81	311	90	20	44	9	2A.1, 2A.2, 2B, 4D, 6A.3
	BLUE	172	173	89	31	332	96	25	12	5	2A.2, 2B, 4D, 6A.3
Adult-plant tests for 175 entries	07MV	160	14	0	1	166	7	1	1	6	5B.2, 7B.1
	07PCFS	139	32	0	4	162	8	5	0	2	6B
	08MV	134	28	1	12	148	14	11	1	3	1D
	08PCFS	129	23	0	23	164	6	4	1	1	–
	08SP	120	30	1	24	171	2	2	0	2	4A, 6B
	09MV	155	20	0	0	158	16	1	0	0	–
	09PCFS	86	53	4	32	156	14	2	3	2	1A.1
	09SP	166	8	0	1	163	9	1	2	2	4A, 5B.3
	10MV	93	59	4	19	82	63	18	7	1	–
	10PCFS	143	17	0	15	129	27	9	10	3	–
	10SP	86	34	2	53	116	37	20	2	1	5B.3
	11MV	160	15	0	0	107	56	12	0	4	5B.3
	11PCFS	142	32	0	1	140	24	10	0	2	3D
	11SP	133	35	0	7	140	25	6	4	1	4B
	12MV	93	81	0	1	95	61	16	3	3	5B.1
	12PCFS	73	45	0	57	130	33	2	10	4	3D, 6A.4
	12SP	102	61	0	12	121	33	11	7	1	2A.2
	13MV	38	134	0	3	120	46	6	2	1	–
	13PCFS	84	42	1	48	128	34	9	4	1	–
	13SP	71	63	1	40	128	41	4	1	3	1D, 4A
	14MV	147	24	3	1	122	35	17	1	2	–
	14PCFS	32	121	0	22	105	48	14	8	0	–
	14SP	21	118	1	35	71	51	35	18	2	7A.1
	15MV	143	20	9	3	140	25	9	1	5	6B
	15PCFS	91	45	1	38	156	4	6	6	1	–
	15SP	71	99	0	5	166	8	1	0	0	–
	16MV	103	58	12	2	120	38	14	3	2	4B
	16PCFS	115	40	0	20	163	6	2	4	4	1A.1, 1A.2, 3B
	16SP	104	57	0	14	156	13	0	6	5	1A.2
	17MV	36	136	0	3	106	51	16	2	3	2A.1, 7B.2
	17PCFS	108	40	0	27	61	63	37	14	2	7B.2
	17SP	82	87	0	6	161	11	0	3	5	7A.1
	BLUE (175)	47	119	9	0	143	29	3	0	2	6A.4

HR, high resistance; MR, moderate resistance; MS, moderately susceptible; HS, highly susceptible; MTA, marker–trait associations; -, not applicable or not detected.

a The numbers are the years (07-21 for 2007–2021), and the letters are for the locations (MV, Mount Vernon; PCFS, Palouse Conservation Field Station; and SP, Spillman Farm near Pullman; BLUE, best linear unbiased estimator across the environments).

b Chromosome (and number) of QTL detected in this study.

For the 175 entries that were also tested from 2007 to 2017, 165 (94.29%) showed moderate to high levels of resistance (BLUE_rDS ≤50%, BLUE_IT ≤5) across the 32 environments (**Figures 1D, E**, **Table 1**; **Supplementary Table S4**). The rDS values under these environments were consistently low, except for some variations in resistance levels in the environments of 10MV, 10SP, 11MV, 14SP, and 17PCFS. These results further demonstrated the durability of the HTAP resistance in most of the entries.

**Figure 2:**
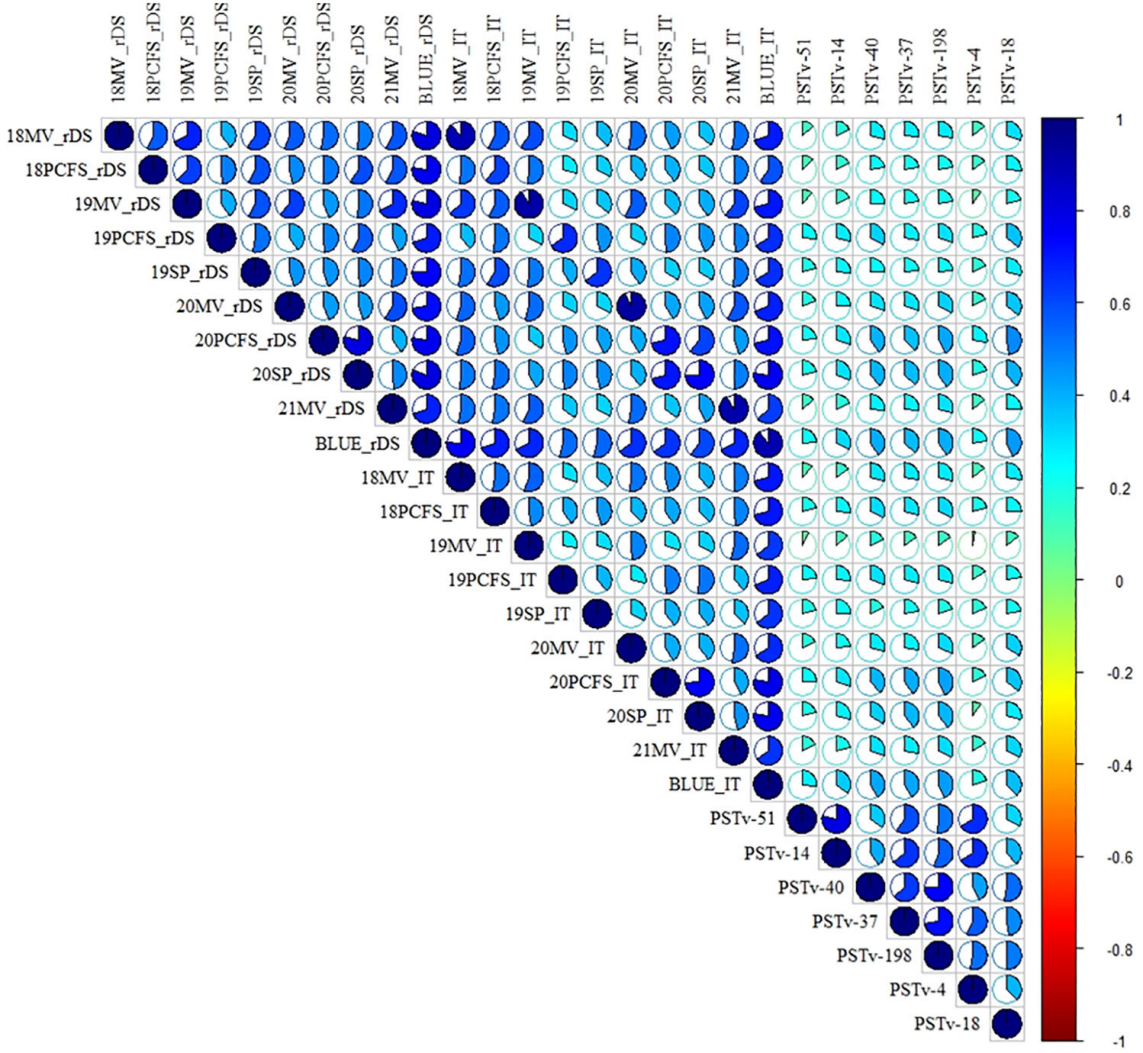
Heatmap of the correlation coefficients of stripe rust responses at the adult-plant and seedling stages of the 465 entries in the whole winter wheat panel. Positive to negative correlations are displayed in blue to red colors. The color intensity and the scale of the ellipse chart are proportional to the correlation coefficients. rDS, relative disease severity; IT, infection type; 18-21, 2018–2021; BLUE, best linear unbiased estimator across all field environments; MV, Mount Vernon; PCFS, Palouse Conservation Field Station; SP, Spillman Farm.

Positive correlations in stripe rust response were observed across all tests (**Figure 2**; **Supplementary Table S5**). In the field adult-plant tests, the correlation coefficients ranged from 0.40 to 0.80 based on the rDS data and ranged from 0.28 to 0.78 based on the IT data across the environments. The correlation coefficients between the rDS and IT data across the environments ranged from 0.28 to 0.91 and usually relatively high within the same environments and relatively low between different environments. In the greenhouse seedling tests, the correlation coefficients were from 0.32 to 0.79 across the different race tests. These correlations were at moderate to high levels. However, the correlation coefficients between the greenhouse seedling tests and the field environments were low, ranging from 0.09 to 0.42 between the IT data in the seedling tests and rDS data in the field tests and from 0.02 to 0.43 between the IT data in the seedling tests and IT data in the field tests. These results indicated that the resistance values observed at the seedling stage under the controlled greenhouse conditions (at low temperatures) and at the adult-plant stage under the field conditions (mostly at high temperatures) are mostly controlled by different genes for different types of resistance. Broad-sense heritability (*H* ^2^) was calculated as 0.89 and 0.92 for the IT and rDS data for the field tests, respectively (**Table 2**), indicating that the resistance, mostly HTAP resistance, detected at the adult-plant stage across the different environments was consistent and highly inheritable.

**Table 2:** Summary of stripe rust infection type (IT) and relative disease severity (rDS, %) data of the winter wheat panel (465 entries) in the tests with seven races of *Puccinia striiformis* f. sp. *tritici* at the seedling stage under the controlled greenhouse conditions and at the adult-plant stages under natural infection of the pathogen in various year-location environments and broad-sense heritability (*H* ^2^) in the field tests.

Trait	Race or environment^a^	Minimum	Maximum	Mean	Stdev	CV	*H* ^2^
Seedling IT in green-house	PSTv-14	1	9	7.64	2.02	0.26	NA
	PSTv-18	1	9	3.73	2.51	0.67	
	PSTv-198	1	9	7.50	2.13	0.28	
	PSTv-37	1	9	7.48	2.27	0.30	
	PSTv-4	1	9	7.64	2.32	0.30	
	PSTv-40	1	9	7.14	2.48	0.35	
	PSTv-51	1	9	7.80	1.97	0.25	
Adult-plant IT in fields	18MV	1	8	3.37	1.95	0.58	0.89
	18PCFS	2	8	3.57	2.23	0.63	
	19MV	2	8	3.35	2.01	0.60	
	19PCFS	0	8	4.75	2.75	0.58	
	19SP	0	8	3.78	2.39	0.63	
	20MV	2	9	3.57	2.28	0.64	
	20PCFS	1	9	4.39	2.78	0.63	
	20SP	2	9	4.97	2.77	0.56	
	21MV	2	9	3.65	2.34	0.64	
	BLUE	1.75	8.38	3.97	1.68	0.42	
Adult-plant rDS in fields	18MV	1	106	25.83	28.58	1.11	0.92
	18PCFS	1	100	9.90	19.49	1.97	
	19MV	2	100	22.04	24.03	1.09	
	19PCFS	0	105	22.50	24.20	1.08	
	19SP	0	108	17.26	19.13	1.11	
	20MV	2	100	21.99	24.79	1.13	
	20PCFS	1	103	24.77	28.94	1.17	
	20SP	0	100	23.25	22.78	0.98	
	21MV	2	100	24.04	26.75	1.11	
	BLUE	1.75	94.50	20.94	18.37	0.88	

Stdev, standard deviation; CV, coefficient of variation; NA, not applicable as the test did not have repeats.

a The environment consists of the year and location: 18-21, the years of 2018–2021; MV, Mount Vernon, Washington; PCFS, Palouse Conservation Field Station; and SP, Spillman Farm near Pullman, Washington; BLUE, best linear unbiased estimator.

### 3.2 Population structure, genetic diversity, and LD

**Figure 3:**
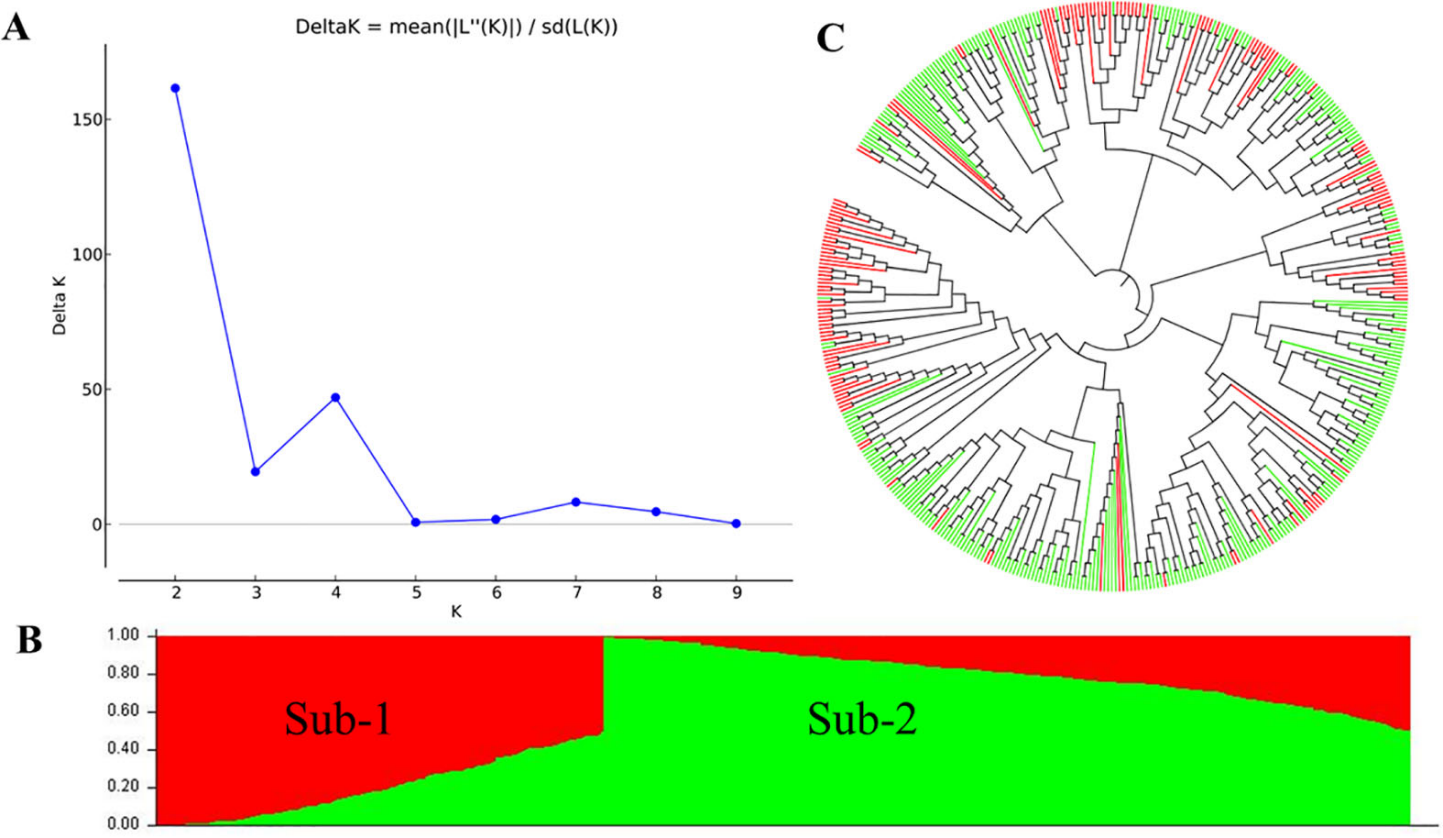
Population structure of the whole winter wheat panel consisting of 465 entries. **(A)** Estimated ΔK of the structure inferred by STRUCTURE HARVESTER. **(B)** Two subpopulations inferred by structural analysis using the software STRUCTURE V2.3.4. The red and green parts represent sub-1 and sub-2, respectively. **(C)** Neighbor-joining phylogenetic tree. The red and green branches represent the sub-1 and sub-2 accessions corresponding to the structural analysis.

After filtering the SNPs with missing data >50% and MAF <0.05, 1,278 polymorphic SNPs from GMS were obtained and used in the analysis of population structures (**Supplementary Table S6**). Based on the results from the STRUCTURE HARVESTER analysis (**Figure 3A**) and STRUCTURE V2.3.4 analysis (**Figure 3B**), the 465 entries were classified into two sub-populations (sub-1 and sub-2). Sub-1 consisted of 165 entries, while Sub-2 had 300, and these results were consistent with the neighbor-joining phylogenetic tree analysis (**Figure 3C**).

**Figure 4:**
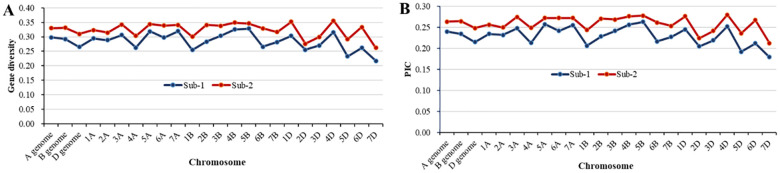
Comparison of the genetic diversity between sub-1 and sub-2. **(A)** Gene diversity and **(B)** polymorphism information content (PIC).

Among the 1,278 polymorphic SNPs, 1,088 with known genomic positions were used to estimate the genetic diversity and LD. The panel had a high gene diversity value of 0.32 and polymorphism information content (PIC) value of 0.26 for the three genomes (**Table 3**). Among the chromosomes, the gene diversity and PIC values were quite similar, ranging from 0.26 on 7D to 0.36 on 5B for gene diversity and from 0.21 on 7D to 0.29 on 5B for PIC. The relatively even coverages of the genomes and chromosomes indicate that the polymorphic SNPs are suitable for GWAS. Regarding the two subpopulations, the gene diversity and PIC values of sub-2 were higher than those of sub-1 across the genomes and individual chromosomes (**Figure 4**). The difference in genetic diversity may be due to the different numbers of entries and geographic origins. The sub-1 entries were mainly from the US Pacific Northwest, while the entries in sub-2 were from numerous countries including other regions of the United States.

**Table 3:** Number of polymorphic SNP markers across the chromosomes and genomes identified through genotyping by multiplex sequencing, gene diversity, and polymorphism information content (PIC) of the winter wheat panel.

Chromosome	No. of SNPs	Gene diversity	PIC
1A	57	0.33	0.26
2A	69	0.32	0.25
3A	63	0.34	0.27
4A	50	0.30	0.25
5A	79	0.35	0.28
6A	61	0.34	0.27
7A	80	0.35	0.27
A genome	459	0.33	0.26
1B	61	0.30	0.24
2B	59	0.33	0.27
3B	68	0.34	0.27
4B	37	0.35	0.28
5B	84	0.36	0.29
6B	54	0.32	0.26
7B	61	0.32	0.25
B genome	424	0.33	0.26
1D	39	0.35	0.27
2D	35	0.29	0.23
3D	31	0.30	0.24
4D	12	0.35	0.28
5D	29	0.28	0.23
6D	26	0.32	0.26
7D	33	0.26	0.21
D genome	205	0.31	0.25
Whole genome	1,088	0.32	0.26

**Figure 5:**
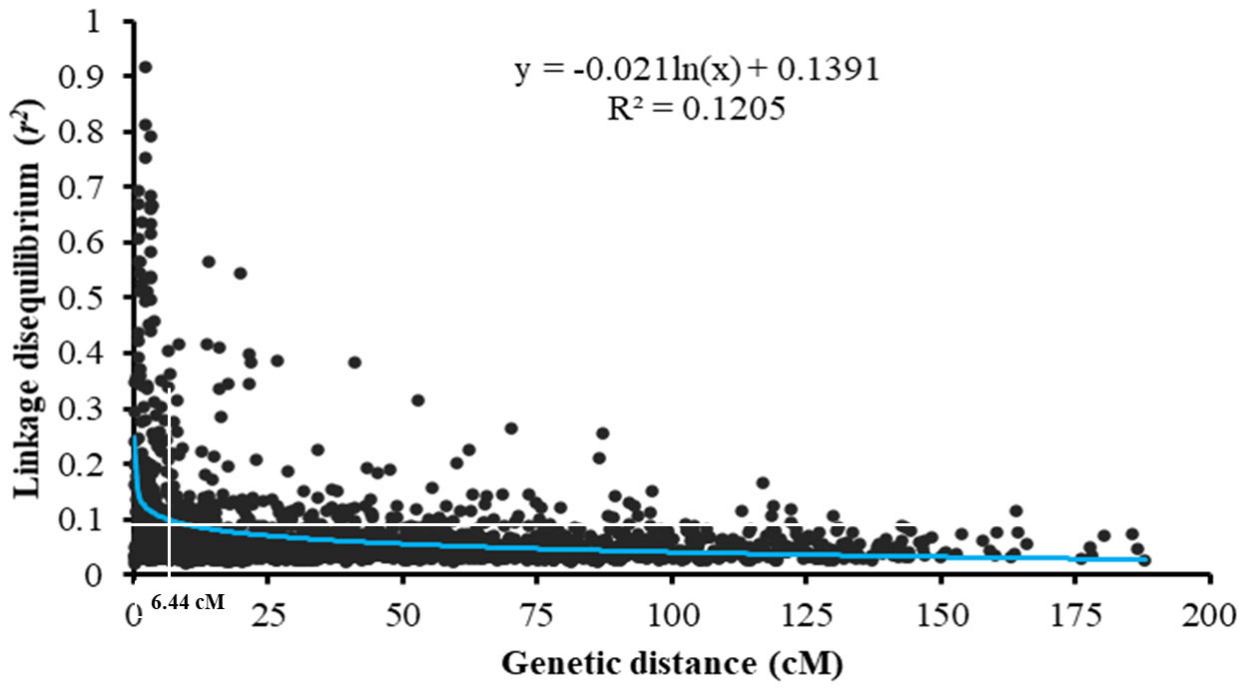
Genome-wide linkage disequilibrium (LD) decays over the genetic distance of the whole winter wheat panel consisting of 465 entries. The blue curve represents the model fitting LD decays. The red line represents the standard critical *r* ^2^ = 0.10 used to establish QTL confidence intervals.

The LD analysis was conducted based on the pairwise squared allele frequency correlations (*r* ^2^) for all intra-chromosomal SNPs. In total, 31,549 pairwise comparisons for the 1,088 SNP markers were used for the LD analysis. A scatter plot and a decay curve of LD *r* ^2^ against the genetic distance are presented in **Figure 5**. With the increasing distance between intra-chromosomal markers, the correlation *r* ^2^ dropped rapidly to 0.10 and then decreased slowly. Thus, 0.10 was used as the critical value for the significance of *r* ^2^, and the distance of 6.44 cM in the curve that intercepted the critical *r* ^2^ was determined as the mean LD decay. Therefore, SNPs significantly associated with stripe rust response in the same chromosome within 6.44 cM were considered in the same QTL.

### 3.3 Significant QTL identified through GWAS analyses

**Figure 6:**
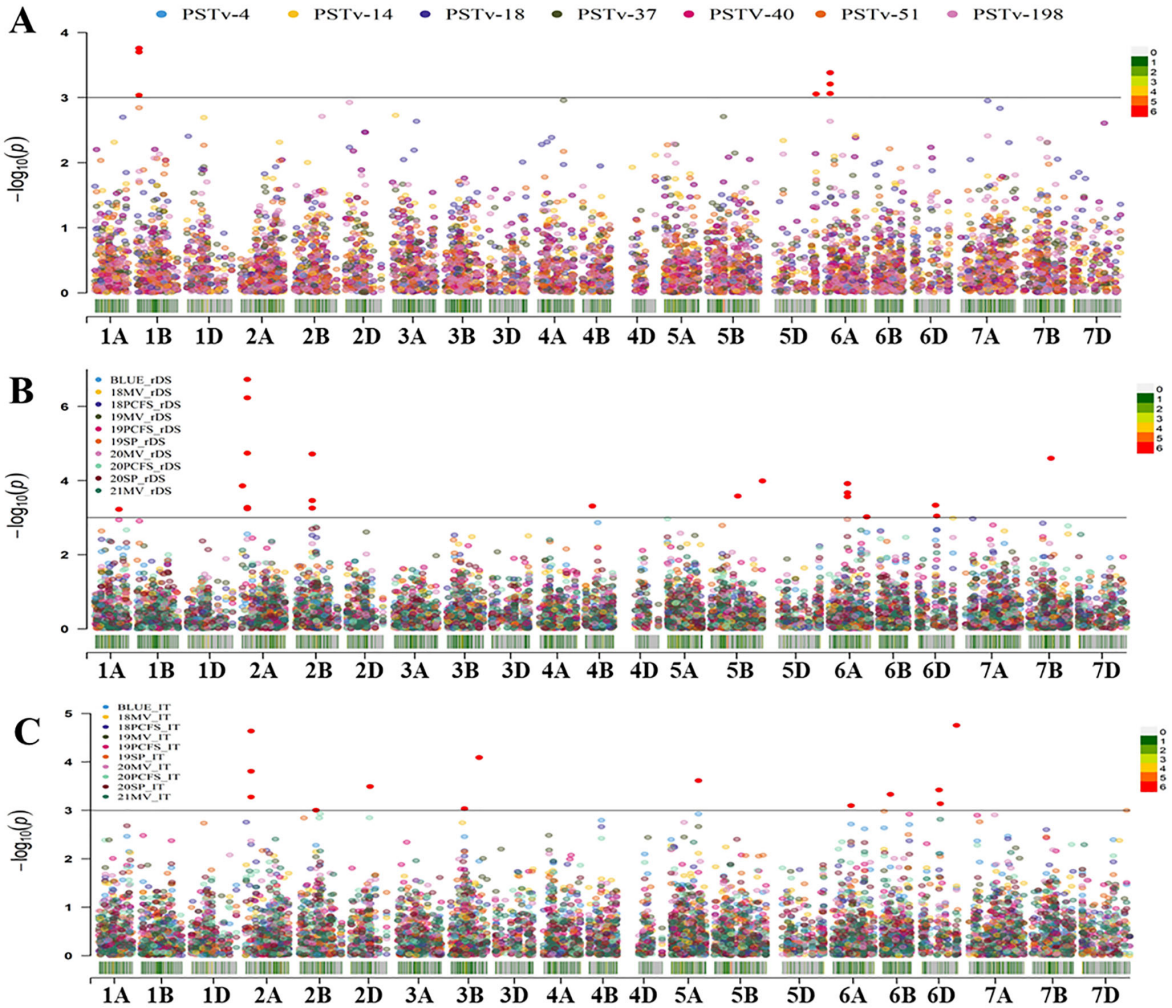
Distribution of the marker–trait associations (MTAs) for stripe rust response of the whole winter wheat panel consisting of 465 entries on the 21 chromosomes based on the infection type data of the seedling tests with seven *Puccinia striiformis* f. sp. *tritici* races **(A)**, relative disease severity (rDS) data **(B)**, and infection type (IT) data **(C)** at the adult-plant stage in various environments (year and location). BLUE, best linear unbiased estimator across all field environments; 18-21, the years 2018–2021; MV, Mount Vernon; PCFS, Palouse Conservation Field Station; SP, Spillman Farm.

Two sets of GWAS were conducted. The first set of analysis was done using 1,278 polymorphic SNPs (MAF ≥ 0.05) and the stripe rust IT and rDS data at the adult-plant stage of 465 wheat entries tested in nine field environments from 2018 to 2021 plus the BLUE data across environments and the seedling IT data tested with seven *Pst* races in the greenhouse. The second set of analysis was done using 1,200 polymorphism SNPs (MAF ≥ 0.05) and the IT and rDS data of 175 entries from 32 environments from 2007 to 2017 plus the BLUE data across the environments. Using the MLM (Q+K) with the significant threshold *P* ≤ 0.001 [-log_10_(*P*) ≥ 3.0], a total of 143 significant MTAs were identified from the two sets of GWAS analyses, and the number of MTA detected in each race test or field environment is given in **Table 1**. The distributions of the MTAs across the 21 chromosomes associated to the IT data from the seedling tests with the seven *Pst* races in the greenhouse and the rDS and IT data from the field environments are illustrated in **Figures 6A–C**, respectively. When the MTAs were detected in two or more race tests/field environments or located within the LD decay distance (6.44 cM), they were treated as a single QTL. Thus, 28 QTL were obtained, and they were on chromosomes 1A (2), 1B, 1D, 2A (2), 2B, 3B, 3D, 4A, 4B, 4D, 5B (3), 5D, 6A (4), 6B, 6D (3), 7A (2), and 7B (2) (**Table 4**). These QTL were named as *QYrWW.wgp-1A.1*, *QYrWW.wgp-1A.2*, *QYrWW.wgp-1B*, *QYrWW.wgp-1D*, *QYrWW.wgp-2A.1*, *QYrWW.wgp-2A.2*, *QYrWW.wgp-2B*, *QYrWW.wgp-3B*, *QYrWW.wgp-3D*, *QYrWW.wgp-4A*, *QYrWW.wgp-4B*, *QYrWW.wgp-4D*, *QYrWW.wgp-5B.1*, *QYrWW.wgp-5B.2*, *QYrWW.wgp-5B.3*, *QYrWW.wgp-5D*, *QYrWW.wgp-6A.1*, *QYrWW.wgp-6A.2*, *QYrWW.wgp-6A.3*, *QYrWW.wgp-6A.4*, *QYrWW.wgp-6B*, *QYrWW.wgp-6D.1*, *QYrWW.wgp-6D.2*, *QYrWW.wgp-6D.3*, *QYrWW.wgp-7A.1*, *QYrWW.wgp-7A.2*, *QYrWW.wgp-7B.1*, and *QYrWW.wgp-7B.2*, respectively. Each of the QTL explained 6%–40% of the phenotypic variation (*R* ^2^).

**Table 4:** QTL significantly associated to stripe rust resistance at the seedling stage tested with races of *Puccinia striiformis* f. sp. *tritici* (*Pst*) and at the adult-plant stage under the natural infection of the pathogen in various field environments (years and locations) in the winter wheat panel.

QTL name	SNP marker	Test stage^a^	Race or field environ._trait^b^	Genetic position (cM)^c^	-log_10_(*P*)	MAF	Allele^d^	*R* ^2e^	Effect	Reported *Yr* genes/QTL^f^	Reference
*QYrWW.wgp-1A.1*	*IWA2995*	Adult	16PCFS_IT	79.75	3.7	0.35	T/**C**	0.14	-0.72	*QYr.sun-1A Yrzhong12-2*	Bariana et al., 2010; Ma et al., 2014
	*IWA1608*	Adult	09PCFS_rDS	82.04	3.8	0.31	T/**G**	0.2	7.47		
*QYrWW.wgp-1A.2*	*IWA6710*	Adult	16PCFS_rDS	99.03	3.3	0.06	T/**G**	0.14	14.69	*QYrww.wgp.1A-2*	Mu et al., 2020
	*IWA6710*	Adult	16SP_rDS	99.03	3.2	0.06	T/**G**	0.23	15.16		
*QYrWW.wgp-1B*	*IWB11262*	Seedling	PSTv-4_IT	9.41	3.8	0.22	**T**/C	0.29	-0.74	*QYr.cau-1BS*	Quan et al., 2013
	*IWB11262*	Seedling	PSTv-14_IT	9.41	3	0.22	**T**/C	0.16	-0.61		
	*IWB11262*	Seedling	PSTv-37_IT	9.41	3.7	0.22	**T**/C	0.22	-0.73		
*QYrWW.wgp-1D*	*IWB15847*	Adult	08MV_IT	47.66	4	0.25	T/**C**	0.11	-0.76	*Qyr.ucw-1D QYrww.wgp.1D-3*	Maccaferri et al., 2015; Mu et al., 2020
	*IWA362*	Adult	13SP_rDS	48.81	3.4	0.42	T/**C**	0.08	-6.03		
	*IWB10919*	Adult	08MV_IT	54.49	3.4	0.19	T/**C**	0.09	-0.81		
*QYrWW.wgp-2A.1*	*IWB14868*	Adult	17MV_IT	3.75*	3.6	0.4	**T**/C	0.22	-0.45	*QYrtm.pau-2A QYr.uga-2AS QYr.ufs-2A QYrva.vt- 2AS QYr.sun-2A QYr.ucw-2A.2 Qyr.wpg-2A.2 QYrSW.wgp-2A.1*	Chhuneja et al., 2008; Hao et al., 2011; Agenbag et al., 2012; Christopher et al., 2013; Bansal et al., 2014; Maccaferri et al., 2015; Naruoka et al., 2015; Liu et al., 2020
	*IWB22615*	Adult	19SP_rDS	3.94	3.9	0.35	**T**/C	0.19	8.52		
	*IWB42693*	Adult	18MV_IT	9.91	3.3	0.23	**A**/C	0.14	0.69		
	*IWB42693*	Adult	18MV_rDS	9.91	4.7	0.23	**A**/C	0.2	12.48		
	*IWB42693*	Adult	18PCFS_rDS	9.91	3.2	0.23	**A**/C	0.12	6.84		
	*IWB42693*	Adult	19MV_IT	9.91	3.8	0.23	**A**/C	0.11	0.77		
	*IWB42693*	Adult	19MV_rDS	9.91	3.3	0.23	**A**/C	0.16	8.57		
	*IWB42693*	Adult	19SP_rDS	9.91	6.2	0.23	**A**/C	0.21	9.84		
	*IWB42693*	Adult	21MV_IT	9.91	4.6	0.23	**A**/C	0.26	0.96		
	*IWB42693*	Adult	21MV_rDS	9.91	6.7	0.23	**A**/C	0.3	13.58		
*QYrWW.wgp-2A.2*	*Yr17-KASP*	Adult	BLUE_rDS	NA	3.2	0.35	**A**/G	0.28	4.05	*Yr17* closely linked markers	Wang and Chen, 2017
	*Yr17-KASP*	Adult	18MV_IT	NA	3.3	0.35	**A**/G	0.14	0.45		
	*Yr17-KASP*	Adult	18MV_rDS	NA	5.4	0.35	**A**/G	0.21	8.855		
	*Yr17-KASP*	Adult	18PCFS_IT	NA	5.3	0.35	**A**/G	0.22	0.672		
	*Yr17-KASP*	Adult	18PCFS_rDS	NA	4.5	0.35	**A**/G	0.13	5.32		
	*Yr17-KASP*	Adult	19MV_IT	NA	5.1	0.35	**A**/G	0.13	0.59		
	*Yr17-KASP*	Adult	19MV_rDS	NA	5.4	0.35	**A**/G	0.18	7.52		
	*Yr17-KASP*	Adult	19SP_rDS	NA	6.2	0.35	**A**/G	0.21	6.47		
	*Yr17-KASP*	Adult	20MV_rDS	NA	3.1	0.35	**A**/G	0.2	5.1		
	*Yr17-KASP*	Adult	21MV_IT	NA	3.7	0.35	**A**/G	0.25	0.55		
	*Yr17-KASP*	Adult	21MV_rDS	NA	7.9	0.35	**A**/G	0.31	9.83		
	*Yr17-KASP*	Adult	12SP_IT	NA	3.2	0.11	**A**/G	0.11	0.78		
*QYrWW.wgp-2B*	*IWB72086*	Adult	BLUE_rDS	91.6	3.3	0.05	A/**G**	0.28	8.52	*QYrid.ui-2B.1*	Chen et al., 2012
	*IWB72086*	Adult	19MV_rDS	91.6	3.5	0.05	A/**G**	0.16	12.36		
	*IWB72086*	Adult	21MV_IT	91.6	3	0.05	A/**G**	0.25	1.04		
	*IWB72086*	Adult	21MV_rDS	91.6	4.7	0.05	A/**G**	0.29	15.41		
*QYrWW.wgp-3B*	*IWB5332*	Adult	16PCFS_IT	74.37	4	0.22	T/**C**	0.14	-0.97	*QYr.uga-3BS.2 QYr.uga-3BS.3*	Hao et al., 2011
	*IWB10937*	Adult	18PCFS_IT	75.23	3	0.36	T/**C**	0.2	0.54		
*QYrWW.wgp-3D*	*IWB64376*	Adult	11PCFS_IT	144.18*	4.2	0.32	**T**/C	0.11	0.43	ND^h^	
	*IWB64376*	Adult	11PCFS_rDS	144.18*	3.6	0.32	**T**/C	0.12	6.4		
	*IWB52592*	Adult	12PCFS_rDS	148.48*	3.2	0.37	A/**G**	0.19	14.38		
*QYrWW.wgp-4A*	*IWB57645*	Adult	08SP_IT	152.71	3.3	0.29	T/**C**	0.19	-1.16	*QYrww.wgp.4A-3*	Mu et al., 2020
	*IWB39715*	Adult	09SP_IT	158.57	3.1	0.17	**T**/C	0.24	0.44		
	*IWB39715*	Adult	13SP_rDS	158.57	3.3	0.17	**T**/C	0.08	9.26		
*QYrWW.wgp-4B*	*IWB59186*	Adult	11SP_IT	41.52	3.2	0.46	**A**/G	0.08	-3.65	New	
	*IWB59186*	Adult	16MV_rDS	41.52	3.1	0.46	**A**/G	0.16	-40.54		
*QYrWW.wgp-4D*	*Kasp856*	Adult	BLUE_IT	NA	3.9	0.05	**A**/G	0.25	-1.14	*Yr46*	Herrera-Foessel et al., 2011
	*Kasp856*	Adult	BLUE_rDS	NA	5.4	0.05	**A**/G	0.3	-14.56		Forrest et al., 2014
	*Kasp856*	Adult	18MV_rDS	NA	3.4	0.05	**A**/G	0.19	-18.36		
	*Kasp856*	Adult	18PCFS_rDS	NA	6.6	0.05	**A**/G	0.15	-19.23		
	*Kasp856*	Adult	19MV_rDS	NA	4.5	0.05	**A**/G	0.17	-18.66		
	*Kasp856*	Adult	20MV_IT	NA	3.2	0.05	**A**/G	0.15	-1.45		
	*Kasp856*	Adult	20MV_rDS	NA	3.7	0.05	**A**/G	0.21	-16.74		
	*Kasp856*	Adult	21MV_rDS	NA	3	0.05	**A**/G	0.28	-15.11		
*QYrWW.wgp-5B.1*	*IWB46807*	Adult	19PCFS_rDS	164.11	3.6	0.36	**T**/C	0.22	-9.3	*QYr.caas-5BL.2 QYr.sun-5B*	Lu et al., 2009; Bariana et al., 2010
	*IWB6746*	Adult	12MV_IT	164.84	3.4	0.22	**T**/C	0.14	0.72		
*QYrWW.wgp-5B.2*	*IWB29548*	Adult	19PCFS_rDS	232.94	4	0.08	**T**/C	0.22	12.69	*QYr.sun-5B*	Bansal et al., 2014
	*IWB29548*	Adult	07MV_IT	232.94	3.1	0.07	**T**/C	0.25	-0.89		
*QYrWW.wgp-5B.3*	*IWA4280*	Adult	10SP_IT	124.06	3	0.1	**T**/C	0.07	-1.94	*QYrSW.wgp-5B.2*	Liu et al., 2020
	*IWA4280*	Adult	10SP_rDS	124.06	3	0.1	**T**/C	0.2	-16.37		
	*IWA4280*	Adult	11MV_IT	124.06	3	0.1	**T**/C	0.06	-0.87		
	*IWA4280*	Adult	11MV_rDS	124.06	3.7	0.1	**T**/C	0.13	-15.92		
*QYrWW.wgp-5D*	*IWA4561*	Seedling	PSTv-51_IT	183.39	3.1	0.29	**T**/C	0.21	0.77	*QYrbr.wpg-5D*	Case et al., 2014
*QYrWW.wgp-6A.1*	*IWB41817*	Seedling	PSTv-4_IT	16.17	3.4	0.15	A/**C**	0.29	-0.63	*QYrex.wgp-6AS QYr.uga-6AS*	Lin and Chen, 2009; Hao et al., 2011
	*IWB41817*	Seedling	PSTv-14_IT	16.17	3.2	0.15	A/**C**	0.16	-0.56		
	*IWB41817*	Seedling	PSTV-40_IT	16.17	3.5	0.15	A/**C**	0.4	-0.61		
	*IWB41817*	Seedling	PSTv-51_IT	16.17	3.1	0.15	A/**C**	0.2	-0.52		
*QYrWW.wgp-6A.2*	*IWB29623*	Seedling	PSTv-14_IT	40.53	3.9	0.29	T**/C**	0.17	-0.81	New	
	*IWB29623*	Seedling	PSTv-51_IT	40.53	3.3	0.29	T**/C**	0.2	-0.7		
*QYrWW.wgp-6A.3*	*IWB22698*	Adult	BLUE_rDS	98.55	3.7	0.25	**A**/C	0.29	5.32	*QYr.cim-6AL* *6AL-QTL*	Lillemo et al., 2008; Lan et al., 2014
	*IWB22698*	Adult	18MV_rDS	98.55	3	0.25	**A**/C	0.19	7.68		
	*IWB22698*	Adult	18PCFS_rDS	98.55	3.9	0.25	**A**/C	0.13	6.33		
	*IWB22698*	Adult	20MV_rDS	98.55	3	0.25	**A**/C	0.2	6.47		
	*IWB22698*	Adult	21MV_IT	98.55	3.1	0.25	**A**/C	0.25	0.62		
	*IWB22698*	Adult	21MV_rDS	98.55	3.6	0.25	**A**/C	0.28	7.62		
*QYrWW.wgp-6A.4*	*IWA6484*	Adult	BLUE_rDS(175)	183.95	3.2	0.41	A/**G**	0.15	-7.86	*QYrww.wgp.6A-2*	Mu et al., 2020
	*IWA6484*	Adult	12PCFS_rDS	183.95	3.1	0.41	A/**G**	0.19	-15.58		
*QYrWW.wgp-6B*	*IWB38887*	Adult	07PCFS_rDS	142.56	3.5	0.49	**T**/C	0.19	4.69	*Qyr.wpg-6B.2*	Naruoka et al., 2015
	*IWA6179*	Adult	15MV_IT	144.27	3.2	0.22	A/**G**	0.14	-0.54		
	*IWA6179*	Adult	15MV_rDS	144.27	3.1	0.22	A/**G**	0.16	-5.52		
	*IWA3224*	Adult	08SP_rDS	150.01	3.2	0.24	**T**/C	0.17	-4.06		
*QYrWW.wgp-6D.1*	*IWB4284*	Seedling	PSTV-40_IT	20.75	3.7	0.18	**A/** C	0.4	-0.64	New	
*QYrWW.wgp-6D.2*	*IWA3624*	Adult	19MV_IT	82.14	3.4	0.22	T/**G**	0.11	-0.55	*QYr.ufs-6D* *QYr.ucw-6D* *QYrSW.wgp-6D*	Agenbag et al., 2012; Maccaferri et al., 2015; Liu et al., 2020
	*IWA3624*	Adult	19MV_rDS	82.14	3.3	0.22	T/**G**	0.16	-6.49		
	*IWA7816*	Adult	20PCFS_IT	87.17	3.1	0.23	**T**/C	0.21	0.8		
	*IWA7816*	Adult	20PCFS_rDS	87.17	3	0.23	**T**/C	0.26	8.08		
*QYrWW.wgp-6D.3*	*IWB5832*	Seedling	PSTV-198_IT	150.56	3.9	0.26	T/**C**	0.21	0.69	6DS-QTL	Zegeye et al., 2014
	*IWB5832*	Seedling	PSTV-51_IT	150.56	3.3	0.26	T/**C**	0.29	0.61		
*QYrWW.wgp-7A.1*	*IWB44935*	Adult	17SP_rDS	155.85	3.1	0.39	**T**/C	0.1	-7.43	New	
	*IWB11533*	Adult	14SP_rDS	156.97	4.5	0.13	A/**G**	0.18	-18.44		
*QYrWW.wgp-7A.2*	IWA8393	Seedling	PSTV-40_IT	164.7	3.1	0.32	**T**/C	0.4	-0.51	New	
*QYrWW.wgp-7B.1*	*IWB41262*	Adult	19PCFS_rDS	93.82	4.6	0.13	A/**C**	0.22	11.73	*Yr39*	Lin and Chen, 2007
	*IWB41262*	Adult	07MV_IT	93.82	3.2	0.09	A/**C**	0.26	-0.85		
*QYrWW.wgp-7B.2*	*IWA320*	Adult	17MV_rDS	106.15	3.1	0.24	A/**G**	0.3	7.34	*QYr.sun-7B QYr.caas-7BL.1*	Bariana et al., 2010
	*IWA320*	Adult	17PCFS_rDS	106.15	3.6	0.24	A/**G**	0.13	12.53		Ren et al., 2012

MAF, minor allele frequency.

a Adult, stripe rust response recorded at the adult-plant stage in the field tests; Seedling, seedling tests with different *Pst* races in the greenhouse.

b 07-21, the years of 2007 to 2021; BLUE, best linear unbiased estimator; MV, Mount Vernon; PCFS, Palouse Conservation Field Station; SP, Spillman; IT, infection type; rDS, relative disease severity (%).

c Genetic distance, the genetic position corresponds to the genetic position in the integration map of Maccaferri et al. (2015), and the marker position with * corresponds to the position in the 90K consensus map of Wang et al. (2014).

d Bold indicates the minor allele; underline indicates the favorable allele.

e Phenotypic variance explained by the significant associated marker.

f New, the presumably new QTL.

h ND, not determined.

Among the 28 QTL, seven were detected at the seedling stage and considered for race-specific ASR and 21 QTL were detected only at the adult-plant stage in the fields and considered as HTAP resistance QTL. Among the seven ASR QTL, *QYrWW.wgp-1B* was detected with races PSTv-4, PSTv-14, and PSTv-37, *QYrWW.wgp-5D* with PSTv-51, *QYrWW.wgp-6A.2* with PSTv-14 and PSTv-51, *QYrWW.wgp-6A.3* with PSTv-4, PSTv-14, PSTV-40, and PSTv-51, *QYrWW.wgp-6D.1* with PSTv-40, *QYrWW.wgp-6D.3* with PSTv-198 and PSTv-51, and *QYrWW.wgp-7A.2* with PSTv-40. Among the 21 HTAP resistance QTL, 12 (*QYrWW.wgp-1A.1*, *QYrWW.wgp-1A.2*, *QYrWW.wgp-1D*, *QYrWW.wgp-3D*, *QYrWW.wgp-4A*, *QYrWW.wgp-4B*, *QYrWW.wgp-5B.3*, *QYrWW.wgp-6A.4*, *QYrWW.wgp-6B*, *QYrWW.wgp-7A.1*, *QYrWW.wgp-7B.1*, and *QYrWW.wgp-7B.2*) were detected with the 175 entries in the 2007–2017 environments, four (*QYrWW.wgp-2B*, *QYrWW.wgp-4D*, *QYrWW.wgp-6A.3*, and *QYrWW.wgp-6D.2*) with the 465 entries in the 2018-2021 environments, and five (*QYrWW.wgp-2A.1*, *QYrWW.wgp-2A.2*, *QYrWW.wgp-3B*, *QYrWW.wgp-5B.1*, and *QYrWW.wgp-5B.2*) with both 175 and 465 entries in the 2007–2021 environments. Two of the QTL were detected to be significantly associated with previously reported *Yr17* (*QYrWW.wgp-2A.2*) and *Yr46* (*QYrWW.wgp-4D*) with *R* ^2^ range of 11%–31% and 11%–26%, respectively (**Table 4**).

### 3.4 Comparison of significant QTL with known *Pst* resistance genes

The 28 QTL were compared with the previously reported *Yr* genes/QTL using the integration map (Wang et al., 2014; Maccaferri et al., 2015). Based on the chromosomal/genome locations, resistance type, and wheat variety origin, 23 QTL were overlapped (6.44 cM) with the previously reported *Yr* genes/QTL. Five QTL (*QYrWW.wgp-4B*, *QYrWW.wgp-6A.2*, *QYrWW.wgp-6D.1*, *QYrWW.wgp-7A.1*, and *QYrWW.wgp-7A.2*) were located far away from the previously reported *Yr* genes/QTL on chromosomes 4B, 6A, 6D, and 7A, and therefore they were considered new in the present study (**Table 4**).

### 3.5 KASP markers developed for eight QTL for HTAP resistance

A total of 10 KASP markers were developed for eight of the QTL for HTAP resistance, and their associations with stripe rust responses identical to their SNPs were confirmed through testing the 465 entries. The primer sequences are shown in **Table 5**. These polymorphic KASP markers should be useful in marker-assisted selections to develop stripe rust-resistant cultivars with the resistance QTL.

**Table 5:** Primer sequences of KASP markers developed from SNP markers significantly associated with eight QTL for high-temperature adult-plant resistance to stripe rust.

QTL name	Physical position (Mb)^a^	KASP marker	Primer sequence (5′–3′)
*QYrWW.wgp-1A.1*	472.17	*IWA2995-A*	GAAGGTGACCAAGTTCATGCTCATCTCCACAGTTGCAATGGT
		*IWA2995-B*	GAAGGTCGGAGTCAACGGATTCATCTCCACAGTTGCAATGGC
		*IWA2995-C*	CTGAGCAGCTGCCCTTACTT
*QYrWW.wgp-1A.2*	518.01	*IWA6710-A*	GAAGGTGACCAAGTTCATGCTAATCTTGCCATTCTTTAGGGGTA
		*IWA6710-B*	GAAGGTCGGAGTCAACGGATTAATCTTGCCATTCTTTAGGGGTC
		*IWA6710-C*	GAAGATACCATTAGTGAAGCAGAAC
*QYrWW.wgp-1D*	32.54	*IWA362-A*	GAAGGTGACCAAGTTCATGCTGGAGTATTGCGATGAGGTGAAT
		*IWA362-B*	GAAGGTCGGAGTCAACGGATTGGAGTATTGCGATGAGGTGAAC
		*IWA362-C*	AGGAGTCTCAGGTATTGATTGATAC
*QYrWW.wgp-1D*	47.85	*IWB10919-A*	GAAGGTGACCAAGTTCATGCTCCTCTTGTGACTTGTGTGGGA
		*IWB10919-B*	GAAGGTCGGAGTCAACGGATTCCTCTTGTGACTTGTGTGGGG
		*IWB10919-C*	TACACCAACTGATCGAGCTAC
*QYrWW.wgp-2B*	69.65	*IWB72086-A*	GAAGGTGACCAAGTTCATGCTGCAGGAAAAATTGCGAGCCA
		*IWB72086-B*	GAAGGTCGGAGTCAACGGATTGCAGGAAAAATTGCGAGCCG
		*IWB72086-C*	TCCAACCGCCAAGCTTTTTG
*QYrWW.wgp-3B*	66.58	*IWB5332-A*	GAAGGTGACCAAGTTCATGCTACCATCATAAGCTCATCGGAAT
		*IWB5332-B*	GAAGGTCGGAGTCAACGGATTACCATCATAAGCTCATCGGAAC
		*IWB5332-C*	GGAACTGACCTGCTTGTCGA
*QYrWW.wgp-3B*	66.87	*IWB10937-A*	GAAGGTGACCAAGTTCATGCTTTAGTGTCAGGATGTAGATTGCATA
		*IWB10937-B*	GAAGGTCGGAGTCAACGGATTTTAGTGTCAGGATGTAGATTGCATG
		*IWB10937-C*	TCTTTCCTTCTCACTGCTTGTCA
*QYrWW.wgp-4A*	700.42	*IWB39715-A*	GAAGGTGACCAAGTTCATGCTAAACCGCTTTCTGGAAGAGT
		*IWB39715-B*	GAAGGTCGGAGTCAACGGATTAAACCGCTTTCTGGAAGAGC
		*IWB39715-C*	CGCCGCCGCTAATTTACAA
*QYrWW.wgp-6A.3*	447.83	*IWB22698-A*	GAAGGTGACCAAGTTCATGCTAGGTGAATTGAGCTGATTGTTGA
		*IWB22698-B*	GAAGGTCGGAGTCAACGGATTAGGTGAATTGAGCTGATTGTTGC
		*IWB22698-C*	ACACGCTGATAACCACGAATAGA
*QYrWW.wgp-6D.2*	178.68	*IWA3624-A*	GAAGGTGACCAAGTTCATGCTCAATATTCACAACCCCACATGTGCA
		*IWA3624-B*	GAAGGTCGGAGTCAACGGATTCAATATTCACAACCCCACATGTGCC
		*IWA3624-C*	AGAGTGCTCAGTAGGCAATCGAC

a The physical positions were referred to the “Chinese Spring” physical map in IWGSC RefSeqV1.0 (International Wheat Genome Sequencing Consortium, 2018).

### 3.6 Frequencies of the *Yr* genes/QTL detected by markers

The presence (+) and absence (-) of the 13 *Yr* genes/QTL identified using previously reported markers and the 28 QTL identified through GWAS in the 456 wheat entries are provided in **Supplementary Table S7**. Entries considered having each of the 41 resistance genes/QTL are listed in **Supplementary Table S8**. The frequencies of the genes/QTL varied from 0.86% (for *Yr36*) to 95.91% (for *QYrWW.wgp-1A.2*). A total of 16 genes/QTL (*Yr36*, *Yr62*, *QYrWW.wgp-6D.3*, *Yr30*, *Yr46*, *QYrWW.wgp-4D*, *Yr16*, *QYrWW.wgp-3B*, *QYrWW.wgp-6A.1*, *QYrWW.wgp-1B*, *Yr59*, *Yr78*, *QYrWW.wgp-6A.2*, *Yr17*, *QYrWW.wgp-2A.2*, and *QYrWW.wgp-6B*) had low frequencies (<10%). There were 17 genes/QTL (*QYrsk.wgp-3BS*, *QYrWW.wgp-1D*, *QYrWW.wgp-5B.3*, *QYrWW.wgp-7A.2*, *QYrWW.wgp-1A.1*, *QYrsk.wgp-4BL*, *QYrWW.wgp-6D.1*, *QYrWW.wgp-6D.2*, *Qyr.wgp-1B.1*, *QYrWW.wgp-2A.1*, *QYrWW.wgp-3D*, *QYrWW.wgp-4A*, *QYrWW.wgp-7B.2*, *QYrWW.wgp-5B.1*, *QYrWW.wgp-7A.1*, *QYrWW.wgp-5D*, and *Yr52*) that had moderate frequencies (13.98%–61.08%). Eight genes/QTL (*QYrWW.wgp-6A.3*, *QYrWW.wgp-6A.4*, *Yr18*, *QYrWW.wgp-4B*, *QYrWW.wgp-2B*, *QYrWW.wgp-7B.1*, *QYrWW.wgp-1A.2*, and *QYrWW.wgp-5B.2*) had relatively high frequencies (74.84%–95.91%).

### 3.7 Pyramiding effect of resistance genes/QTL

**Figure 7:**
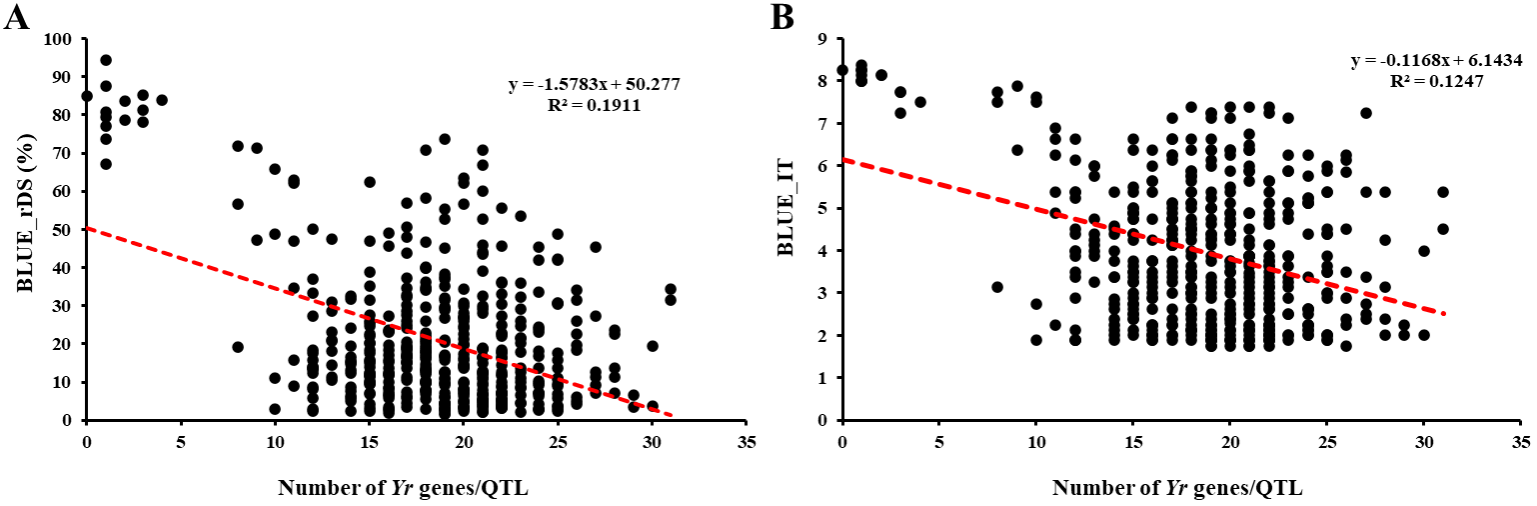
Effects of the number of Yr genes/QTL on stripe rust responses **(A)** for relative disease severity (rDS) and **(B)** for infection type (IT) in the 465 winter wheat entries. BLUE, best linear unbiased estimator.

The number of resistance genes/QTL in each entry based on the marker haplotypes was used to assess the pyramiding effect of resistant genes/QTL on stripe rust response. For the resistance observed in the field tests, the BLUE value of IT and rDS values across nine environments were regressed against the numbers of resistant genes/QTL. Since most of the 465 entries have HTAP resistance, with 74.19% of the BLUE_IT lower than 5% and 92.04% of the BLUE_rDS lower than 50%, the distributions of the entries were mainly concentrated in the resistant side, while very few entries were scattered in the susceptible side (**Figure 7**). The number of resistance genes/QTL present in an entry ranged from 0 to 31, and more than 90% of the entries have 11–27 of the favorable alleles (**Figure 7**; **Supplementary Table S7**). With the number of resistance genes/QTL increasing, the stripe rust phenotype (BLUE_rDS and BLUE_IT) was gradually decreasing, indicating that more genes/QTL improved the resistance to stripe rust.

## 4 Discussion

Stripe rust is a serious disease that threatens wheat production in the world (Stubbs, 1985; Chen, 2005, 2020; Wellings, 2011). In the United States, the disease has caused frequent epidemics in the regions west of the Rocky Mountains and has become a major disease in the regions east of the mountain range (Chen, 2007; Chen et al., 2010). This is mainly due to the virulence changes within the pathogen population and the new isolates with significant adaptation to the warm climates (Chen et al., 2002; Chen, 2005, 2020; Milus et al., 2009). To mitigate the problem, it is essential to continuously identify and utilize new resources of stripe rust resistance, especially durable types of resistance. In the present study, a panel of 465 winter wheat entries presumably with HTAP resistance was used to identify stable and potentially new QTL for stripe rust resistance through GWAS and determine the presence of previously reported *Yr* genes or QTL for HTAP resistance through marker-assisted detection.

The 465 entries were tested in different field environments for many years, especially the first part of 175 entries that have been tested for stripe rust response since 2007. A large number of entries with stable resistance that could be used in breeding programs were identified. The 465 entries were also tested at the seedling stage with seven contemporary *Pst* races in the present study. Since these entries were selected with HTAP resistance based on previous greenhouse seedling and field adult-plant tests, most of them should be susceptible at the seedling stage but resistant at the adult-plant stage. The results of the present study prove the significant levels of HTAP in most of the entries. The consistent resistant phenotypes of most entries across different environments indicate their durable resistance.

The 465 entries were genotyped using the GMS platform (Ruff et al., 2020). Previously, Liu et al. (2020) and Mu et al. (2020) used the platform in their studies of US spring and winter wheat panels through GWAS and identified 37 and 51 genes/QTL, respectively. In the present study, we used the same approach, but much more years of field data and identified 28 QTL in the winter wheat HTAP resistance panel. Among the 28 QTL, 21 conferred HTAP resistance and seven for ASR. After comparing with the previously reported *Yr* genes/QTL, five QTL (*QYrWW.wgp-4B*, *QYrWW.wgp-6A.2*, *QYrWW.wgp-6D.1*, *QYrWW.wgp-7A.1*, and *QYrWW.wgp-7A.2*) are newly identified loci for stripe rust resistance. The relationships of the QTL detected in this study and the previously reported *Yr* genes/QTL are discussed below.

Four QTL were identified in homoeologous group 1, namely, *QYrWW.wgp-1A.1*, *QYrWW.wgp-1A.2*, *QYrWW.wgp-1B*, and *QYrWW.wgp-1D*. Except for *QYrWW.wgp-1B*, an ASR locus, the other three QTL were all HTAP loci detected in the field environments, and each of them explained 8% to 29% of phenotypic variation. On the group 1 chromosomes, many genes/OTL for stripe rust resistance have been reported. Ma et al. (2014) reported a race-specific resistance gene on chromosome 1A, provisionally named *Yrzhong12-2*. Its most closely linked marker, *Xcfd2129*, was 1.31 and 3.60 cM away from the association markers *IWA2995* and *IWA1608* of *QYrWW.wgp-1A.1* identified in the present study, respectively. As the distance between the two loci was within the LD decay distance 6.44 cM, they are likely located in the same chromosomal region. However, *QYrWW.wgp-1A.1* confers HTAP resistance, while *Yrzhong12.2* is a race-specific resistance gene. The relationship of *QYrWW.wgp-1A.1* and *Yrzhong12-2* needs to be determined by further studies. Bariana et al. (2010) reported an APR locus in the Australian cultivar Janz, *QYr.sun-1A*, which is adjacent to *IWA2995* and *IWA1608*. Therefore, *QYr.sun-1A* and *QYrWW.wgp-1A.1* should be the same QTL. *QYrWW.wgp-1A.2* was significantly associated with *IWA6710* in two environments (16PFS_rDS and 16SP_rDS), and it was also mapped to chromosome 1A. In a previous study, Mu et al. (2020) identified an APR QTL, *QYrww.wgp.1A-2*, also significantly associated with *IWA6710* in a winter wheat panel. The same marker indicates that *QYrWW.wgp-1A.2* and *QYrww.wgp.1A-2* are the same QTL. On chromosome 1B, *QYrWW.wgp-1B* was an ASR locus identified with races PSTv-4, PSTv-14, and PSTv-37 and significantly associated with *IWB11262* with the negative effect of seedling IT (-0.74, -0.61, and -0.73, respectively). Quan et al. (2013) reported that *QYr.cau-1BS* affected the latent period of *Pst* infection at the seedling stage and mapped it to the interval *gwm374-gwm264-barc194* on chromosome 1B. On the integrated map, *gwm374* is only 0.64 cM away from *IWB11262* associated to *QYrWW.wgp-1B*. Thus, *QYrWW.wgp-1B* and *QYr.cau-1BS* are most likely the same locus. On chromosome 1D, *QYrWW.wgp-1D* was significantly associated with *IWA362*, *IWB15847*, and *IWB10919* at the position of 47.66–55.49 cM on chromosome 1D. Maccaferri et al. (2015) reported a locus on chromosome 1D that was significantly associated with *IWA980*, and Mu et al. (2020) reported *QYrww.wgp.1D-3* to be significantly associated with *IWA3446*. On the integrated map, *IWA980* and *IWA3446* were located at 49.26 and 45.09 cM on chromosome 1D, similar to the position of *QYrWW.wgp-1D*. Therefore, these three QTL are likely the same.

Three QTL for HTAP resistance were detected in homoeologous group 2, namely, *QYrWW.wgp-2A.1*, *QYrWW.wgp-2A.2*, and *QYrWW.wgp-2B*. *QYrWW.wgp-2A.1* was significantly associated with *IWB14868*, *IWB22615*, and *IWB42693* on the short arm of chromosome 2A in nine field environments. In fact, there was a hot spot on chromosome 2AS, on which many genes/QTL for stripe rust resistance were reported, including *QYrtm.pau-2A* (Chhuneja et al., 2008), *QYr.uga-2AS* (Hao et al., 2011), *QYr.ufs-2A* (Agenbag et al., 2012), *QYrva.vt-2AS* (Christopher et al., 2013), *QYr.sun-2A* (Bansal et al., 2014), *QYr.ucw‐2A.2* (Maccaferri et al., 2015), *Qyr.wpg-2A.2* (Naruoka et al., 2015), and *QYrSW.wgp-2A.1* (Liu et al., 2020). These QTL were reported in an interval of 5.70–13.40 cM on the integrated map, which is within the confidence interval of *QYrWW.wgp-2A.1*. Among them, *QYrtm.pau-2A* was derived from einkorn wheat (*T. monococcum*) line pau14087, *QYrSW.wgp-2A.1* was an ASR locus (Liu et al., 2020), while the other loci were all APR loci. Though they all fall in a hot spot of stripe rust resistance on chromosome 2A, they still have differences. Therefore, further studies are needed to determine the relationship among these loci. *QYrWW.wgp-2A.2* was detected at the adult-plant stage by the closely linked KASP molecular marker *Yr17-Kasp* of *Yr17*. *Yr17* is an ASR gene, but the KASP marker was only detected at the adult-plant stage in the field experiments. This is consistent with the results of Liu et al. (2020). In fact, *Yr17* has a closely linked HTAP resistance gene, *YrM1225*, on chromosome 2AS (Li et al., 2023). Therefore, *QYrWW.wgp-2A.2* detected by the *Yr17-Kasp* marker should be *YrM1225*. On chromosome 2B, *QYrWW.wgp-2B* conferred HTAP resistance and was identified with *IWB72086* in the 19MV and 21MV and BLUE environments. The ASR gene *Yr31* (Jighly et al., 2015), ASR locus *QYrlu.cau-2BS1* (Guo et al., 2008), and the APR locus *QYrid.ui-2B.1* (Chen et al., 2012) were close to *QYrWW.wgp-2B* based on their marker locations on the integrated map. Based on the type of resistance, *QYrWW.wgp-2B* is likely the same as *QYrid.ui-2B.1*, which was identified for HTAP resistance from the Idaho winter wheat breeding line IDO444 (Chen et al., 2012).

*QYrWW.wgp-3B* and *QYrWW.wgp-3D* were identified in homoeologous group 3. *QYrWW.wgp-3B* was detected at 74.37–75.23 cM on chromosome 3B of the integrated map with *IWB5332* and *IWB10937* in the 16PCFS and 18PCFS environments. On chromosome 3B, *QYr.uga-3BS.2* and *QYr.uga-3BS.3* were reported as two minor loci from AGS 2000 in a recombinant inbred population with a 2.4-cM interval (Hao et al., 2011), which could be considered the same locus based on the confidence interval (6.44 cM) of the present study. Thus, these two QTL are likely the same as *QYrWW.wgp-3B*. Another resistance gene, *Yrwh2*, was located at 72.95–75.78 cM of the integrated map, also within the confidence interval of *QYrWW.wgp-3B*, but the resistance type was different, indicating a different locus (Zhou et al., 2014a). Since the genetic position of *QYrWW.wgp-3D* was not found on the integrated map, its relationship with previously reported *Yr* genes/QTL could not be determined.

Three HTAP resistance QTL (*QYrWW.wgp-4A*, *QYrWW.wgp-4B*, and *QYrWW.wgp-4D*) were identified in homoeologous group 4. *QYrWW.wgp-4A* was detected with *IWB57645* (152.71 cM) and *IWB39715* (158.57 cM) on the integrated map, which is overlapped with *QYrww.wgp.4A-3* detected with *IWA559* (154.26 cM) in another winter wheat panel (Mu et al., 2020). Therefore, *QYrWW.wgp-4A* and *QYrww.wgp.4A-3* should be the same. *QYrWW.wgp-4B* was detected at 41.52 cM on chromosome 4B and was found to be a new resistance locus based on its unique position from the previously reported *Yr* genes/QTL reported on chromosome 4B. *QYrWW.wgp-4D* was identified with KASP marker *KASP856* in multiple environments (BLUE_IT, BLUE_rDS, 18MV_rDS, 18PCFS_rDS, 19MV_rDS, 20MV_IT, 20MV_rDS, and 21MV_rDS) on chromosome 4D. This QTL had a relatively large PVE value (15%–30%) and a negative effect value (IT corresponds to -1.14 to -1.45; rDS corresponds from -14.56 to -19.23). *KASP856* was developed for the pleiotropic APR gene *Yr46/Lr67* (for resistance to stripe rust and leaf rust) by Forrest et al. (2014). Therefore, *QYrWW.wgp-4D* should be *Yr46/Lr67.* The frequency of this resistance gene in the HTAP resistance panel was only 2.15% (**Supplementary Table 8**). Although the marker was also used in previous studies, Liu et al. (2020) and Mu et al. (2020) did not detect the gene in their panels consisting of 616 spring wheat and 857 winter wheat entries, respectively, mostly from the United States. The present study indicates that this gene has been started to be used in breeding programs.

Four HTAP QTL (*QYrWW.wgp-5B.1*, *QYrWW.wgp-5B.2*, *QYrWW.wgp-5B.3*, and *QYrWW.wgp-5D*) were identified in homoeologous group 5. *QYrWW.wgp-5B.1* was detected with *IWB46807* (164.11 cM) and *IWB6746* (164.84 cM) on the integrated map, which overlapped with *QYr.sun-5B* detected with *wPt-3030* (158.56 cM) from Janz (Bariana et al., 2010) and *QYr.caas-5BL.2* detected with *gwm604* (166.70 cM) (Lu et al., 2009), indicating them as the same QTL. *QYrWW.wgp-5B.2* was significantly associated with *IWB29548* (232.94 cM) and was approximately 68 cM apart from *QYrWW.wgp-5B.1*. Bansal et al. (2014) reported that *QYr.sun-5B* was closely linked to *wPt-0837* and located at 226.95 cM of the integrated map, which was overlapped with the position of *QYrWW.wgp-5B.2*. Bansal et al. (2014) found that *QYr.sun-5B* was far away from *QYr.sun-5B* and *QYr.caas-5BL.2* reported by Bariana et al. (2010) and Lu et al. (2009), which was consistent with the results of the present study. *QYrWW.wgp-5B.3* was an HTAP resistance QTL detected in the SP and MV field environments and overlapped with an ASR QTL *QYrSW.wgp-5B.2* detected in a spring wheat panel (Liu et al., 2020). Due to the different resistance types, these QTL should be different, but their genetic relationship needs further studies. *QYrWW.wgp-5D* was detected with *IWA4561* (183.39 cM) for seedling reaction to PSTv-51 in the present study, and *QYrbr.wpg-5D* was detected between *Xbarc144* and *Xwmc765* (179.39–183.55 cM) for seedling reaction to PST-114 (Case et al., 2014). Based on their similar chromosomal locations, these two QTL should be the same.

Four ASR QTL (*QYrWW.wgp-6A.1*, *QYrWW.wgp-6A.2*, *QYrWW.wgp-6D.1*, and *QYrWW.wgp-6D.3*) and four HTAP resistance QTL (*QYrWW.wgp-6A.3*, *QYrWW.wgp-6A.4*, *QYrWW.wgp-6B*, and *QYrWW.wgp-6D.2*) were detected in homoeologous group 6. Lin and Chen (2009) reported a major QTL (*QYrex.wgp-6AS*) linked with *Xgwm334* and *Xwgp56*. Hao et al. (2011) reported a minor QTL (*QYr.uga-6AS*) linked with DArT markers *wPt-671561* and *wPt-7840* and adjacent to SSR markers *Xgwm459* and *Xgwm334.* In the present study, the significant association marker *IWB41817* (16.17 cM) of *QYrWW.wgp-6A.1* was 3.78, 1.83, and 1.04 cM away from *wPt-7840*, *Xgwm459*, and *Xgwm334* on the integrated map, respectively, indicating that they are in the same position on chromosome 6A. However, *QYrWW.wgp-6A.1* is an ASR locus. Further studies are needed to determine the relationship between these QTL. *QYrWW.wgp-6A.2* was identified with races PSTv-14 and PSTv-51 and significantly associated with *IWB29623.* This locus should be new since no other genes were reported near the region. The significant association marker *IWB22698* (98.55 cM) of *QYrWW.wgp-6A.3* was 4.21 and 6.13 cM away from the flanker markers *gwm356* and *barc3* of *QYr.cim-6AL* (Lan et al., 2014) and *6AL-QTL* (Lillemo et al., 2008), respectively. Based on the close positions, these three QTL are likely the same. *QYrWW.wgp-6A.4* was significantly associated with *IWA6484* (183.95 cM) and was 0.72 cM apart from the significant association marker *IWA214* for *QYrww.wgp.6A-2* identified in the previous US winter wheat germplasm panel (Mu et al., 2020). *QYrWW.wgp-6B* was significantly associated with three markers (*IWB38887*, *IWA6179*, and *IWA3224*) at 142.56–150.01 cM on chromosome 6B. *Qyr.wpg-6B.2* was significantly associated with *IWA3222* (150.00 cM) on the long arm of chromosome 6B in a US Pacific Northwest winter wheat panel (Naruoka et al., 2015), which is close to the location of *QYrWW.wgp-6B*, indicating that these two QTL are the same. *QYrWW.wgp-6D.1* was an ASR locus identified with PSTv-40 and significantly associated with *IWB4284* (20.75 cM) at the end of the 6D short arm. *QYrWW.wgp-6D.1* should be a new locus since no other genes were reported near the region. *QYrWW.wgp-6D.2* was detected at the 82.14–87.17 cM position of chromosome 6D, which is adjacent to *QYr.ufs-6D* (Agenbag et al., 2012), *QYr.ucw-6D* (Maccaferri et al., 2015), and *QYrSW.wgp-6D* (Liu et al., 2020), and therefore these QTL should be the same. *QYrWW.wgp-6D.3* was an ASR locus identified with races PSTv-51 and PSTv-198 and significantly associated with *IWB5832* at 150.56 cM of the integrated map. Zegeye et al. (2014) reported a ASR QTL (*wsnp_Ex_c62371_62036044*) located at 155.56 cM on the integrated map of chromosome 6DS, which was located within the LD decay distance (6.44 cM). Therefore, these loci are likely the same.

Four APR QTL (*QYrWW.wgp-7A.1*, *QYrWW.wgp-7A.2*, *QYrWW.wgp-7B.1*, and *QYrWW.wgp-7B.2*) were detected in homoeologous group 7. Zegeye et al. (2014) reported an ASR QTL located at 145.14 cM on the integrated map of chromosome 7A, which was more than 10 cM away from the HTAP resistance QTL *QYrWW.wgp-7A.1* (155.85–156.97 cM) and ASR gene *QYrWW.wgp-7A.2* (164.7 cM) identified in the present study. Therefore, *QYrWW.wgp-7A.1* and *QYrWW.wgp-7A.2* should be new QTL for stripe rust resistance. *QYrWW.wgp-7B.1* (*IWB41262*) and *QYrWW.wgp-7B.2* (*IWA320*) were 12.33 cM apart on the chromosome 7B integrated map and were named as two different QTL. The significant association marker *IWB41262* of *QYrWW.wgp-7B.1* was 0.67 cM away from one of the flanking markers (*gwm131*) of *Yr39* (Lin and Chen, 2007), indicating that *QYrWW.wgp-7B.1* is likely *Yr39*. The significant association marker *IWA320* of *QYrWW.wgp-7B.2* was 2.92 cM away from flanker marker *wPt-8106* of *QYr.caas-7BL.1* (Ren et al., 2012) and 2.65 cM away from the peak marker *wPt-3723* of *QYr.sun-7B* (Bariana et al., 2010), indicating that these three QTL are likely the same. Interestingly, Ren et al. (2012) reported that *Yr39* and *QYr.caas-7BL*.1 should be the same or closely linked loci. Through comparative analysis in the present study, we found that these loci were in a hot spot region of chromosome 7BL and were relatively close to each other, so further studies were needed to determine their relationships.

As the present study was aimed to identify genes/QTL associated to HTAP resistance to stripe rust and GWAS might miss resistance alleles either present at very high or very low frequencies, we tested the 465 entries with molecular markers for 13 *Yr* genes/QTL. Among the 13 *Yr* genes/QTL identified using previously reported markers, only *Yr17*-indicated HTAP locus and *Yr46* were detected, as discussed above, while the remaining 11 HTAP loci were not detected through GWAS. The remaining 11 genes/QTL were found at either very high or very low frequencies. *Yr18* was found in 410 (88.17%) entries. The high frequency was expected as *Yr18* has been found to be widespread in wheat landraces and cultivars throughout the world (Lagudah et al., 2009; Krattinger et al., 2011), but with frequencies that were much higher than those found in the US spring and winter wheat cultivars and breeding lines (Liu et al., 2020; Mu et al., 2020). The frequency differences might be due to the different entries of the panels. It was a little surprising that *Yr52* was found in 284 (61.08%) entries. *Yr52* conferring a high level of HTAP resistance to stripe rust was first identified in wheat germplasm accession PI 183527 (Ren et al., 2012), which was originally from India and deposited in the USDA-ARS National Small Grains Collection (NSGC) in 1949 (http://www.ars-grin.gov/cgi-bin/npgs/acc/search.pl?accid=PI?183527). The high frequency was not too different from the frequency detected among 74 US wheat germplasms (Ren et al., 2012). These results indicate that the gene has been widely used in developing contemporary cultivars and breeding lines as well as is widely presented in landraces (**Supplementary Table S8**). Three QTL, *Qyr.wgp-1B.1*, *QYrsk.wgp-4BL*, and *QYrsk.wgp-3BS*, which were previously identified in the US Pacific Northwest wheat cultivars (Naruoka et al., 2015; Liu et al., 2019c), were identified in 27.74%, 23.01%, and 13.98%, respectively. The results indicate that these QTL have been used in breeding programs not only in the Pacific Northwest but also in other regions of the United States. Six HTAP resistance genes, *Yr59* (6.45%), *Yr78* (6.45%), *Yr16* (2.37%), *Yr30* (2.15%), *Yr62* (1.94%), and *Yr36* (0.86%), had frequencies below 10% in the HTAP-resistant winter wheat panel (**Supplementary Table S8**), indicating that these genes have not been widely used in US wheat breeding programs, and they should be used in future breeding efforts to diversify HTAP resistance genes. The 13 genes/QTL as well as those identified from the GWAS should be valuable to develop wheat cultivars with durable resistance to stripe rust. As found in the present study and previous studies (Liu et al., 2020; Mu et al., 2020), pyramiding several HTAP resistance can enhance the level of the durable type of resistance. In the present study, we developed 10 KASP markers for eight QTL. Further efforts should be taken to develop KASP for other QTL to be used in marker-assisted selection to incorporate the useful QTL into new wheat cultivars in various regions.

## 5 Conclusions

In the present study, 465 HTAP-resistant winter wheat entries were tested to identify stripe rust resistance sources and genes. After testing with the seven predominant or most virulent *Pst* races at the seedling stage under low temperatures, 16 entries resistant to all the tested races were identified, making them excellent resources for use in developing new cultivars with stripe rust resistance. The 465 entries were also tested in nine field environments at the adult-plant stage under natural infection conditions. As a result, 172 entries were found to have stably high resistance, including the 16 entries identified in the seedling tests. Combining the stripe rust response and SNP genotype data, 28 QTL were identified in the HTAP-resistant winter wheat panel, including seven for ASR and 21 for HTAP resistance. After comparing with the previously reported *Yr* genes/QTL, five QTL, *QYrWW.wgp-4B*, *QYrWW.wgp-6A.2*, *QYrWW.wgp-6D.1*, *QYrWW.wgp-7A.1*, and *QYrWW.wgp-7A.2*, were identified as new loci for stripe rust resistance. A total of 10 polymorphic KASP markers were developed for eight randomly selected HTAP resistance QTL. Further studies are needed to develop KASP markers for other QTL identified in the present study without KASP markers, especially for the five new QTL. In addition to the QTL identified through GWAS, 13 HTAP resistance genes/QTL were detected using their molecular markers reported in previous studies. Except for two genes, these genes/QTL were not detected in the GWAS analyses. The frequencies of all genes/QTL identified in the present study were determined in the HTAP-resistant winter wheat panel. This study provides winter wheat entries with stable ASR and HTAP resistance, information on QTL, their effects, and frequencies, and user-friendly KASP markers to breeding programs to develop new resistant cultivars to improve the control of stripe rust.

## Authors’ note

Mention of trade names or commercial products in this publication is solely for the purpose of providing specific information and does not imply recommendation or endorsement by the US Department of Agriculture. USDA is an equal opportunity provider and employer.

## Data Availability

The datasets presented in this study can be found in online repositories. The names of the repository/repositories and accession number(s) can be found in the article/**Supplementary Material**.

## Supplementary material

Supplementary Table 4 Information on the 175 US winter wheat entries and their stripe rust data in various locations from 2007 to 2017 (note: four entries showing seedling stage resistance to all the seven races of *Puccinia striiformis* f. sp. *tritici* are highlighted in green).

Supplementary Table 7 Presence (+) and absence (-) of 13 high-temperature adult-plant (HTAP) resistance *Yr* genes/QTL and 28 QTL for all-state or HTAP resistance detected through GWAS in the HTAP panel of 465 winter wheat entries.
